# Paclitaxel-induced inhibition of NSCLC invasion and migration via RBFOX3-mediated circIGF1R biogenesis

**DOI:** 10.1038/s41598-024-51500-1

**Published:** 2024-01-08

**Authors:** Zhanyu Xu, Liping Zheng, Shikang Li

**Affiliations:** 1 Department of Thoracic and Cardiovascular Surgery, The First Affiliated Hospital of Guangxi Medical University, Nanning, 530021 Guangxi Zhuang Autonomous Region People’s Republic of China; 2 Department of Anesthesia Catheter Room, The First Affiliated Hospital of Guangxi Medical University, Nanning, 530021 Guangxi Zhuang Autonomous Region People’s Republic of China

**Keywords:** Non-small-cell lung cancer, Chemotherapy

## Abstract

We previously reported that circIGF1R is significantly downregulated in non-small cell lung cancer (NSCLC) cells and tissues. It inhibits cancer cell invasion and migration, although the underlying molecular mechanisms remain elusive. The invasion and migration of NSCLC cells was analyzed by routine in vivo and in vitro functional assays. Fluorescent in situ hybridization, luciferase reporter assay, RNA pull-down assay and RNA immunoprecipitation (RIP) assay were performed to explore the molecular mechanisms. Mechanism of action of paclitaxel-induced RBFOX3-mediated inhibition of NSCLC invasion and migration was investigated through in vitro and in vivo experiments.Our study reveals that circIGF1R acts as a Competing Endogenous RNA (ceRNA) for miR-1270, thereby regulating Van-Gogh-like 2 (VANGL2) expression and subsequently inhibiting NSCLC cell invasion and migration via the Wnt pathway. We also found that RNA binding protein fox-1 homolog 3 (RBFOX3) enhances circIGF1R biogenesis by binding to IGF1R pre-mRNA, which in turn suppresses migration and invasion in NSCLC cells. Additionally, the chemotherapeutic drug paclitaxel was shown to impede NSCLC invasion and migration by inducing RBFOX3-mediated circIGF1R biogenesis.RBFOX3 inhibits the invasion and migration of NSCLC cells through the circIGF1R/ miR-1270/VANGL2 axis, circIGF1R has the potential to serve as a biomarker and therapeutic target for NSCLC.

## Introduction

Lung cancer ranks first among all malignancies globally in terms of mortality rates^1^. Non-small cell lung cancer (NSCLC) accounts for 85% of all lung cancer cases^2^, and is usually diagnosed at the advanced metastatic stage since the early stages are asymptomatic. Only 15%-20% of the patients with advanced NSCLC benefit from conventional treatment, and although molecular-based targeted therapies and immunotherapy have shown encouraging results, the 5-year survival rate is a dismal 10–15%^3^. In order to develop more effective strategies against advanced NSCLC, it is crucial to elucidate the mechanisms underlying metastasis and identify novel therapeutic targets.

Circular RNAs (circRNA) are non-coding, single-stranded closed circular non-coding RNA molecules^4^ that were previously considered transcriptional noise, but their regulatory functions have since been recognized. Studies increasingly show that circRNAs regulate cancer cell migration, apoptosis, cell cycle and chemoresistance, and their stable structure and specific expression patterns make them ideal promising tumor diagnostic markers^5–7^. CircSOBP suppresses amoeboid migration by acting as a miR-141-3p sponge and modulating MYPT1/p-MLC2 axis, thereby preventing PCa cell migration and invasion^8^. Circ-HMGA2 induces lung adenocarcinoma cell metastasis and epithelial-mesenchymal transition by modulating the miR-1236-3p/ZEB1 axis^9^. Furthermore, circNDUFB2 inhibits NSCLC progression by stimulating anti-tumor immunity and destabilizing IGF2BPs^10^. Research indicates that circHERC1 plays a pivotal role in promoting NSCLC cell invasion and metastasis, primarily through the miR-142-3p/HMGB1 axis, and it also activates the MAPK/ERK and NF-κB pathways, leading to increased cell proliferation and decreased apoptosis^11^. In another notable study, the circTLCD4-RWDD3, packaged in EVs through SUMOylated hnRNPA2B1, has been identified as a key facilitator of lymph node metastasis, marking it as a potential therapeutic target^12^. Additionally, circGUCY1A2 has been shown to suppress lung adenocarcinoma development by interacting with the miR-200c-3p/PTEN axis, opening avenues for new therapeutic approaches^13^.However, previous studies mainly focused on the function of circRNAs as miRNA sponges^14^, and only a few have explored the relationship between circRNAs biogenesis and chemoresistance of tumor cells. There is a need to explore the role of circRNAs in NSCLC in order to uncover novel mechanisms of cancer progression and develop effective diagnostic and therapeutic strategies.

In our previous study^15^, we identified a tumor suppressor circIGF1R that is located on chromosome 15q26.3 and consists of IGF1R exon2. Back splicing of circIGF1R was successfully confirmed using Sanger sequencing. CircIGF1R is expressed at low levels in NSCLC tissues and cells, and its ectopic expression inhibited cellular invasion and migration in vitro. In the current study, we found that circIGF1R upregulates VANGL2 by sponging miR-1270, and inhibits the invasion and migration of NSCLC cells by targeting the downstream Wnt pathway. Furthermore, the splicing factor RBFOX3 binds to the precursor mRNA (pre-mRNA) of IGF1R and increases biogenesis of circIGF1R. Finally, the inhibitory effect of paclitaxel on NSCLC cells was mechanistically linked to the induction of RBFOX3 and increased circIGF1R biogenesis. This study, therefore, sheds light on novel molecular mechanisms underpinning NSCLC progression and opens avenues for targeted therapeutic strategies.

## Results

### circIGF1R directly sponges miR-1270 in NSCLC cells

**Figure 1:**
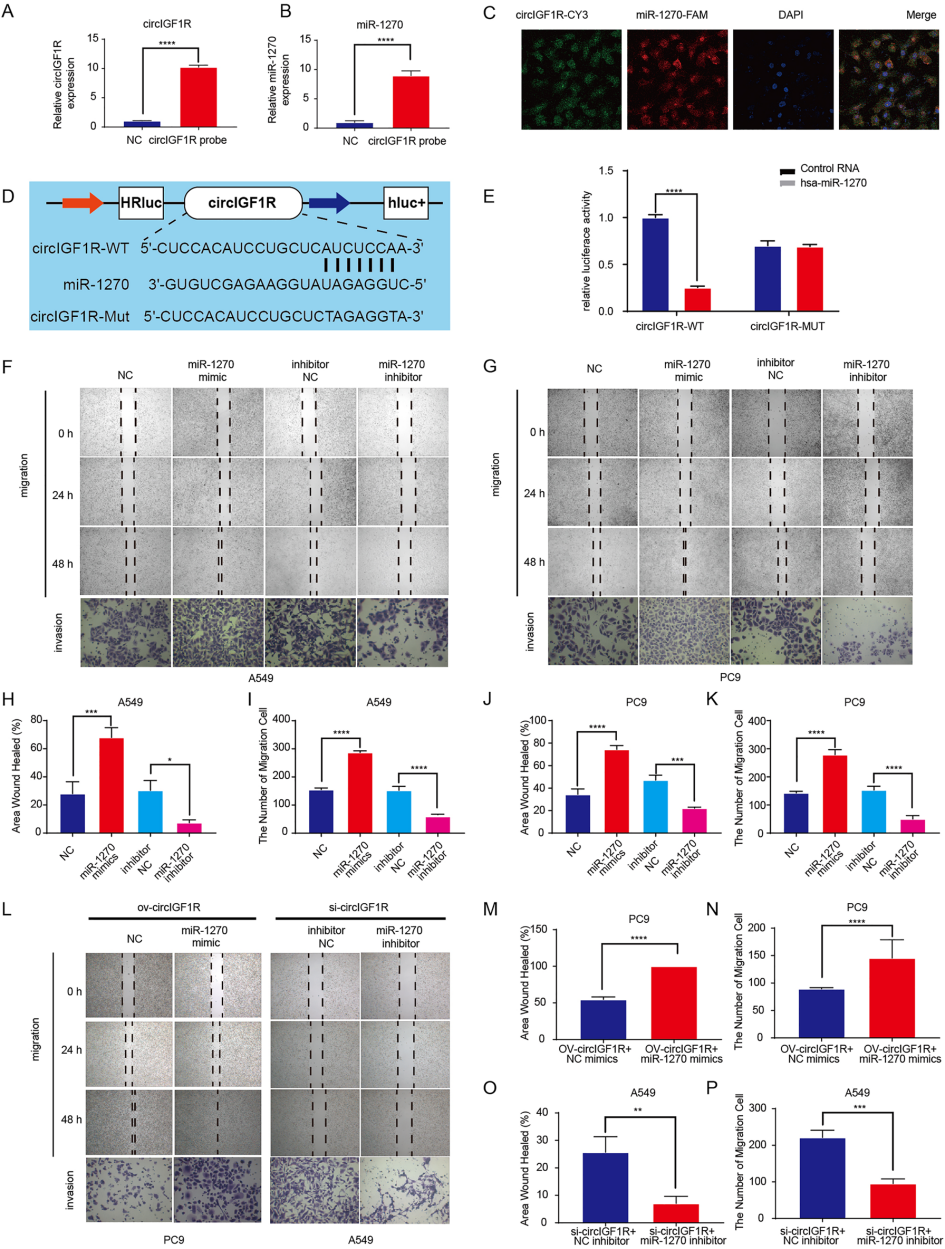
circIGF1R sponged miR-1270 in NSCLC cells. (**A**,**B**) A549 cells were subjected to RNA pull-down using a biotin-labeled circIGF1R probe, followed by qRT-PCR to assess the enrichment of circIGF1R and miR-1270. (**C**) FISH images showing the subcellular localization of miR-1270 and circIGF1R. circIGF1R is stained green (cy3), miR-1270 is labeled red (FAM), and the nuclei are labeled blue (DAPI). (**D**) The wild-type (WT) and mutant (Mut) circIGF1R binding sites of miR-1270 luciferase reporter vectors. (**E**) WT-circIGF1R or Mut-circIGF1R and miR-1270 mimics were co-transfected into A549 cells, and a luciferase reporter gene assay was used to evaluate luciferase activity. (**F**,**G**) Wound healing assay and Transwell migration assay results of A549 and PC9 cells co-transfected with miR-1270 mimic and miR-1270 inhibitor. (H–K) Percentage of migrating and invading cells as calculated in the above assays. (L-P) The invasion and migration of NSCLC cells co-transfected with ov-circIGF1R, si-circIGF1R, miR-1270 mimics or inhibitors. negative control (NC); Inhibitor NC:A549/PC9 + miR-1270 inhibitor negative control. *p < 0.05, **p < 0.01, ***p < 0.001, ****p < 0.0001.

In previous study, we predicted circIGF1R and miR-1270 target binding sequences using bioinformatics, and found a negative correlation between their expression levels in NSCLC cell lines. Consistent with these findings, biotin-coupled RNA pull-down revealed the presence of miR-1270 and circIGF1R in the experimental but not in the control group (Fig. 1A,B). In addition, cytoplasmic co-localization of the fluorescently-labeled circIGF1R and miR-1270 in A549 cells (Fig. 1C), and luciferase reporter assay in A549 cell lines transfected with circIGF1R (Fig. 1D,E) or miR-1270 mimic further confirmed the direct interaction of circIGF1R and miR-1270. The NSCLC cells transfected with ectopic cicrIGF1R showed significantly lower migration and invasion in vitro, whereas si-circIGF1R had the opposite effect. In contrast, miR-1270 mimic promoted the malignant phenotype of the A549 and PC9 cell lines, and the miR-1270 inhibitor had the opposite effect (Fig. 1F–K). Co-transfection of miR-1270 mimic and ov-circIGF1R partially reversed the inhibitory effect of the latter in PC9 cells, whereas cotransfection of miR-1270 inhibitor and si-circIGF1R partially reversed the promoting effect of the latter in A549 cells (Fig. 1L–P). Taken together, circIGF1R acts as a tumor suppressor in NSCLC by sponging off miR-1270.

### CircIGF1R inhibits the malignant phenotype of NSCLC cells by regulating the miR-1270/VANGL2 axis

**Figure 2:**
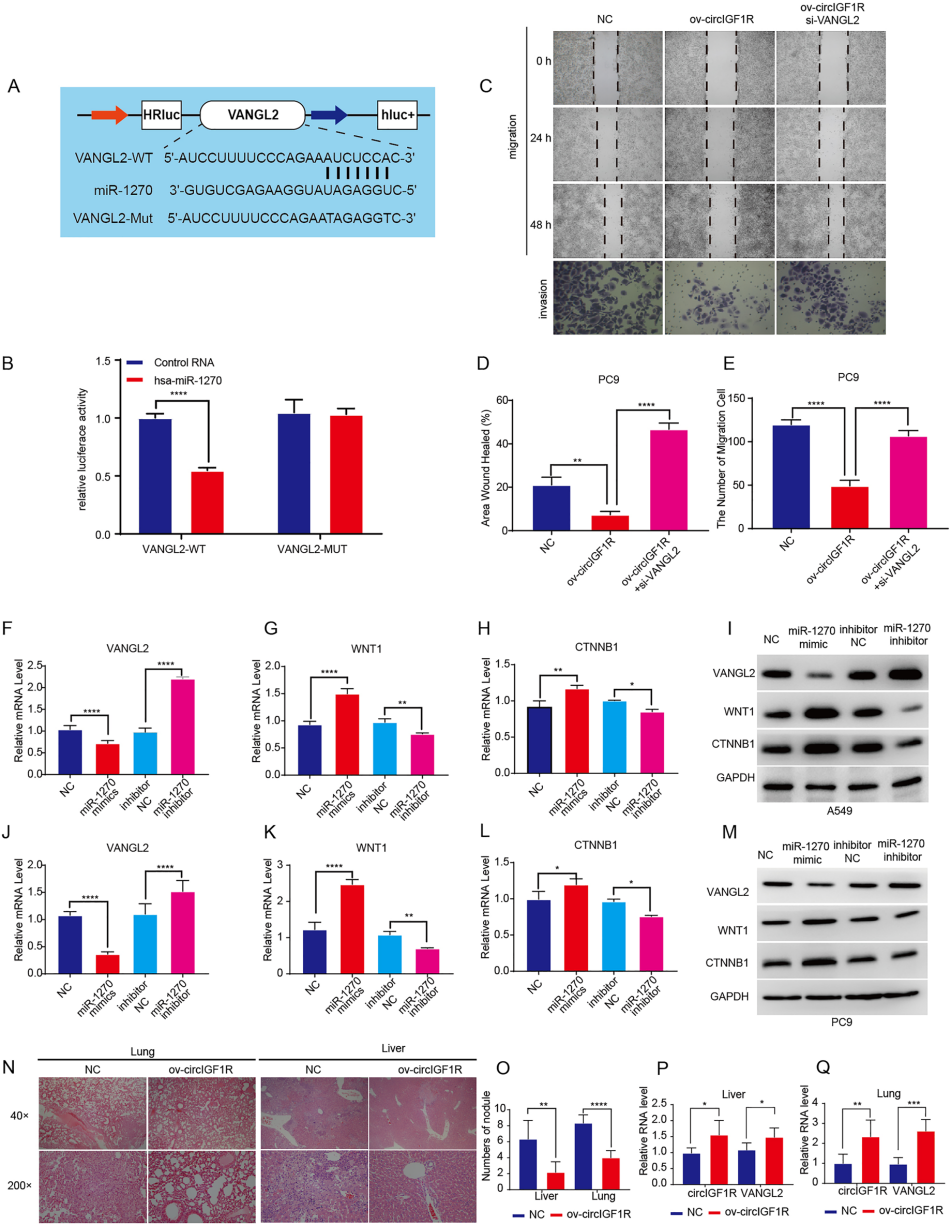
miR-1270 directly targeted VANGL2 (**A**) The wild-type (WT) and mutant (Mut) miR-1270 binding sites in VANGL2 luciferase reporter vectors. (**B**) WT-VANGL2 or Mut-VANGL2 and miR-1270 mimics were co-transfected into A549 cells, and a luciferase reporter gene assay was used to evaluate luciferase activity. (**C–E**) The invasion and migration abilities of PC9 cells co-transfected with ov-circIGF1R and si-VANGL2 were monitored by Transwell and wound healing assays respectively. (**F–M**) The relative expression levels of VANGL2 and downstream WNT-pathway factors in A549 and PC9 cells transfected with the miR-1270 mimics or inhibitors. (**N**) Representative images of H&E stained lung and liver metastatic nodules from mice inoculated with ov-circIGF1R A549 cells (magnification, × 40, × 200). (**O**) Number of lung and liver metastatic nodules in the indicated groups 7 weeks after inoculation. (**P**,**Q**) CircIGF1R and VANGL2 mRNA levels in the lung and liver metastatic nodules. negative control (NC); Inhibitor NC:A549/PC9 + miR-1270 inhibitor negative control. *p < 0.05, **p < 0.01, ***p < 0.001, ****p < 0.0001.

We previously showed that overexpression of circIGF1R up-regulated VANGL2 mRNA and protein in A549 and PC9 cell lines, silencing circIGF1R expression had the opposite effect. The luciferase reporter assay further showed that miR-1270 repressed VANGL2 transcription by binding to its 3′ untranslated region (UTR) (Fig. 2A,B). Also, over-expression of circIGF1R impaired the inhibitory properties of VANGL2 on malignant properties of PC9 cells (Fig. 2C–E). Furthermore, over-expression of miR-1270 markedly suppressed the expression of VANGL2 in A549 and PC9 cells (Fig. 2F,J). To validate the regulation of miR-1270, we evaluated the expression of VANGL2 gene targets including β-catenin1 (CTNNB1) and Wnt family member 1 (WNT1), in A549 and PC9 cells. Our tests revealed an inverse relationship between the aforementioned genes and VANGL2 levels in NSCLC (Fig. 2G-I, K-M).

In the nude mouse models of lung and liver metastasis (Fig. 2N), overexpression of circIGF1R significantly decreased the number of metastatic nodules (Fig. 2O). The expression levels of circIGF1R and VANGL2 in these metastatic nodules were consistent with the in vitro observations (Fig. 2P,Q). Taken together, circIGF1R upregulates VANGL2 by sponging miR-1270, which inhibits NSCLC progression.

### Identification of circIGF1R interacting proteins

**Figure 3:**
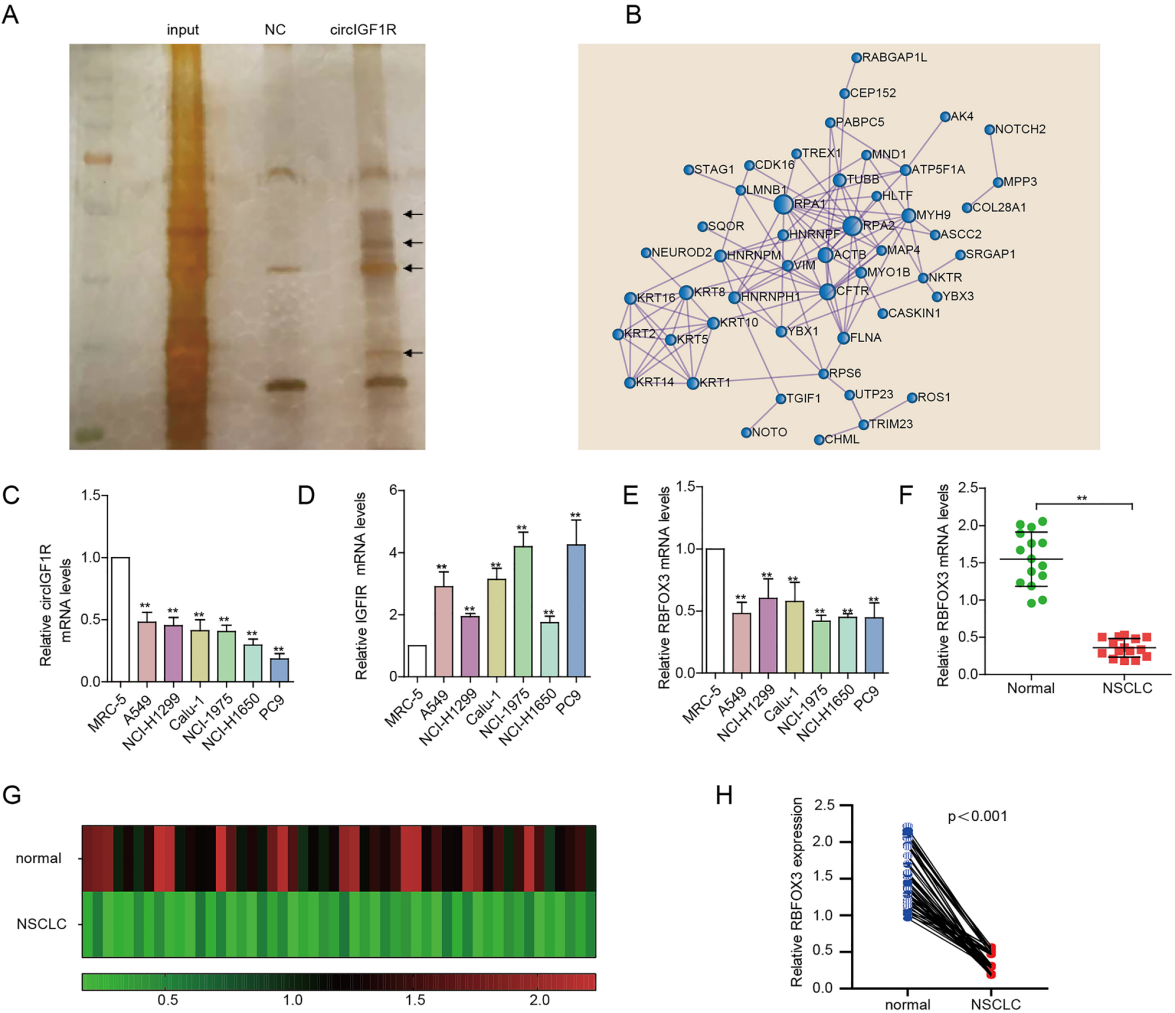
Circ-IGF1R interacts with proteins in NSCLC. (**A**) Silver staining showing proteins pulled down from A549 cell lysates by biotin-labeled NC and circIGF1R. (**B**) Protein–protein interaction network of circIGF1R-binding proteins. (**C–E**) The relative expression levels of circIGF1R and the IGF1R, RBFOX3 mRNAs in NSCLC and lung normal cell lines. (**F**) RBFOX3 mRNA levels in 15 paired NSCLC and para-cancerous tissues. (**G**,**H**) RBFOX3 mRNA levels in 50 paired NSCLC and para-cancerous tissues. **p < 0.01.

We next screened for the circIGF1R-interacting proteins in NSCLC with the RNA pull-down assay using three biotin-labeled probes (same sequence as circIGF1R but without the ability to form circRNA) specific for the back-splice region of circIGF1R. CircIGF1R showed strong interaction with 87 proteins in the A549 cells (Fig. 3A, Supplementary Table S4). The protein–protein interaction (PPI) network of the circRNA-bound proteins is shown in Fig. 3B. Gene ontology (Supplementary Fig. S1A) revealed that these proteins regulate expression of genes via the JAK-STAT signaling pathway following Interleukin-12 stimulation, cellular events in response to growth factors, TOR signaling and DNA biosynthetic process. Thus, circIGF1R interacts with multiple proteins in NSCLC cells, which likely affects pathways involved in cancer progression.

### RBFOX3 is downregulated in NSCLC tissues and correlates with slow tumor progression

To find the binding proteins that splice the IGF1R mRNA to generate circIGF1R, we analyzed the functions of each protein using the Uniprot website. Six proteins with RNA splicing function, including YBX1, HNRNPM, HNRNPH1, HNRNPF, RBFOX3 and FUS, were identified. Interestingly, while circIGF1R was expressed at low levels in NSCLC cell lines (A549, NCI-H1299, Calu-1, NCI-1975, PC9 and NCI-H1650) compared with to the normal lung epithelial cell line MRC-5 (Fig. 3C), the parental gene was significantly upregulated in NSCLC cells as opposed to the MRC-5 cell line (Fig. 3D). In addition, YBX1 and FUS were upregulated in NSCLC cells relative to the MRC-5 cells (Supplementary Fig. S1B, S1C), HNRNPH1, HNRNPF (Supplementary Fig. S1D, S1E) and RBFOX3 (Fig. 3E) were downregulated in the NSCLC cells, whereas HNRNPM expression levels were similar in the NSCLC and normal lung cells (Supplementary Fig. S1F). To further validate these results, we next analyzed HNRNPH1, HNRNPF and RBFOX3 mRNA levels in 15 paired NSCLC and para-cancerous tissues. HNRNPH1 and HNRNPF were similarly expressed in the NSCLC and para-cancerous tissues (Supplementary Fig. S1G, S1H), whereas RBFOX3 expression was significantly downregulated in the tumors (Fig. 3F). In addition, the expression level of RBFOX3 was also found to be lower in 50 NSCLC clinical samples compared to the paired normal tissues (Fig. 3G,H). Therefore, we analyzed RBFOX3 further using A549 and PC9 cell lines with respectively high and low relative expression of circIGF1R.

### RBFOX3 increases circIGF1R biogenesis by binding to IGF1R pre-mRNA and inhibits NSCLC cell invasion and migration in vitro

**Figure 4:**
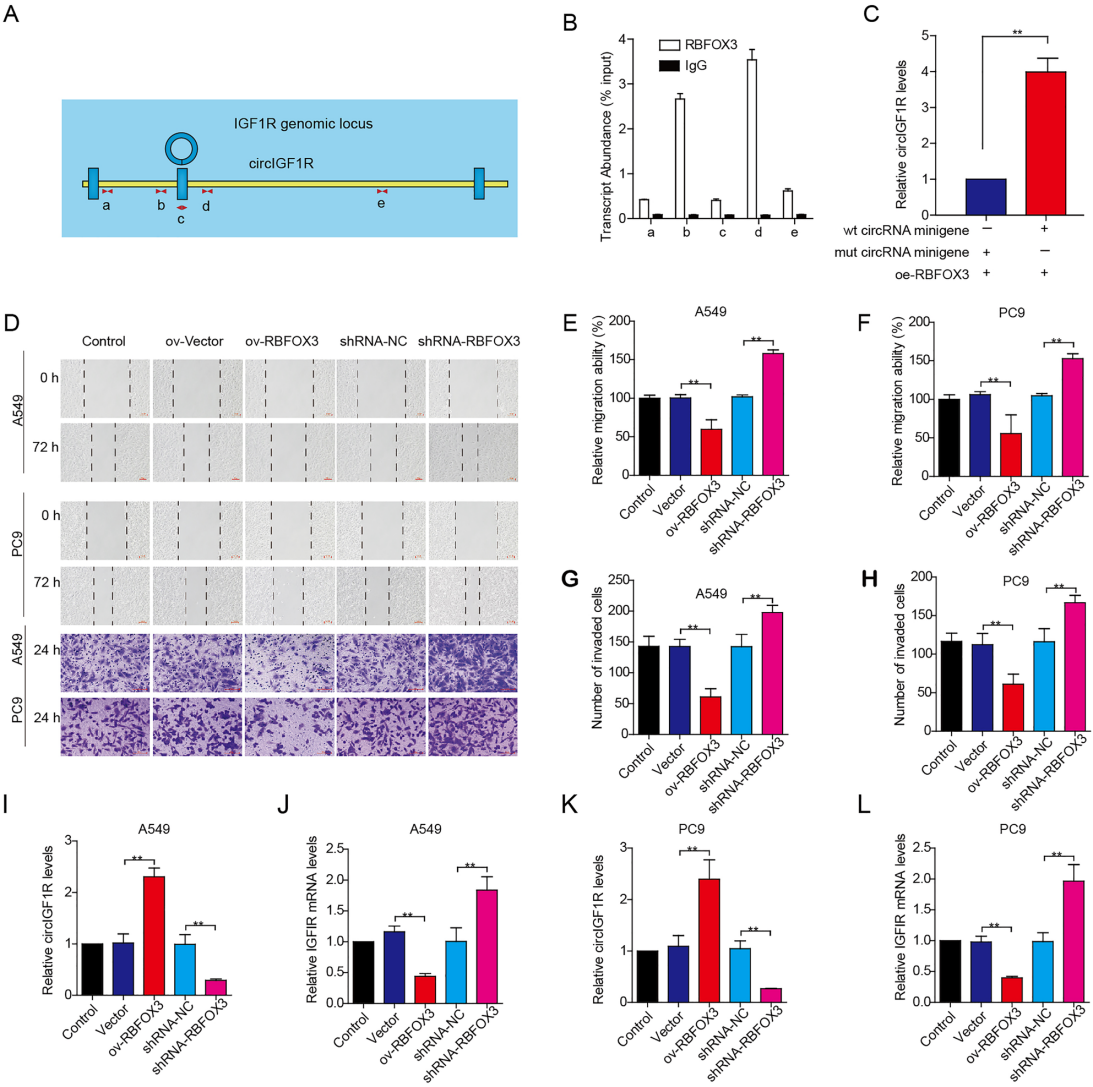
RBFOX3 binds to pre-mRNA of IGF1R in A549 cells. (**A**) Schematics of primer design. (**B**) RIP assay. (**C**) The minigenes were expressed in HEK293T cells and assayed for circIGF1R formation by qPCR. (**D**) Wound healing assay and Transwell migration assay to detect migration of ov-RBFOX3 and sh-RBFOX3 transfected A549 and PC9 cells. (**E–H**) Percentage of invading and migrating A549 and PC9 cells. (**I–L**) circIGF1R and IGF1R mRNA levels. **p < 0.01.

Our previous study showed that circIGF1R is formed by cyclization of the second exon of IGF1R. To verify whether RBFOX3 binds to the pre-mRNA of IGF1R, we predicted their binding sites using the catRAPID website and found that both shared the TGTGCGG sequence. There were 8 common sequences in the pre-mRNA of IGF1R, and we selected the intra-loop (c), near-loop (a, b) and far-loop (d, e) of circIGF1R for the subsequent RIP experiments (Fig. 4A). As shown in Fig. 4B, RBFOX3 bound strongly to the pre-mRNA flanking the near circIGF1R cyclization site (Fig. 4B). To assess the role of RBFOX3 in circRNA biogenesis, we designed 4 primers based on the circIGF1R exon 2 region and the double-flanked intron region containing the RBFOX3 splice site (Intron1 and Intron2), and constructed WT-circIGF1R minigene plasmids. The MUT-circIGF1R minigene plasmids were then constructed by mutating the predicted binding site sequences of both intron regions. Co-transfection of the respective minigene plasmids with the RBFOX3-overexpression plasmid showed that RBFOX3 produced significantly higher levels of circIGF1R from the WT compared to the MUT minigene (Fig. 4C). Furthermore, the protein and mRNA levels of RBFOX3 were significantly overexpressed in A549 and PC9 cells following transfection with the ov-RBFOX3 overexpression plasmid and significantly lower after transfection with the sh-RBFOX3 interference plasmid, indicating high transfection efficiency (Supplementary Figure S2). A549 and PC9 transfected with the ov-RBFOX3 overexpression plasmid showed markedly lower migration and invasion (Fig. 4D–H), which coincided with the significant increase in circIGF1R and decrease in IGF1R mRNA levels (Fig. 4I-L). RBFOX3 knockdown on the other hand enhanced invasion and migration of the NSCLC cells, decreased the expression of circIGF1R, and increased IGF1R mRNA levels. Taken together, RBFOX3 inhibits the migration and invasion ability of PC9 cells and A549 cells by increasing biogenesis of circIGF1R.

### Paclitaxel inhibits NSCLC invasion and migration by inducing RBFOX3-dependent biogenesis of circIGF1R

**Figure 5:**
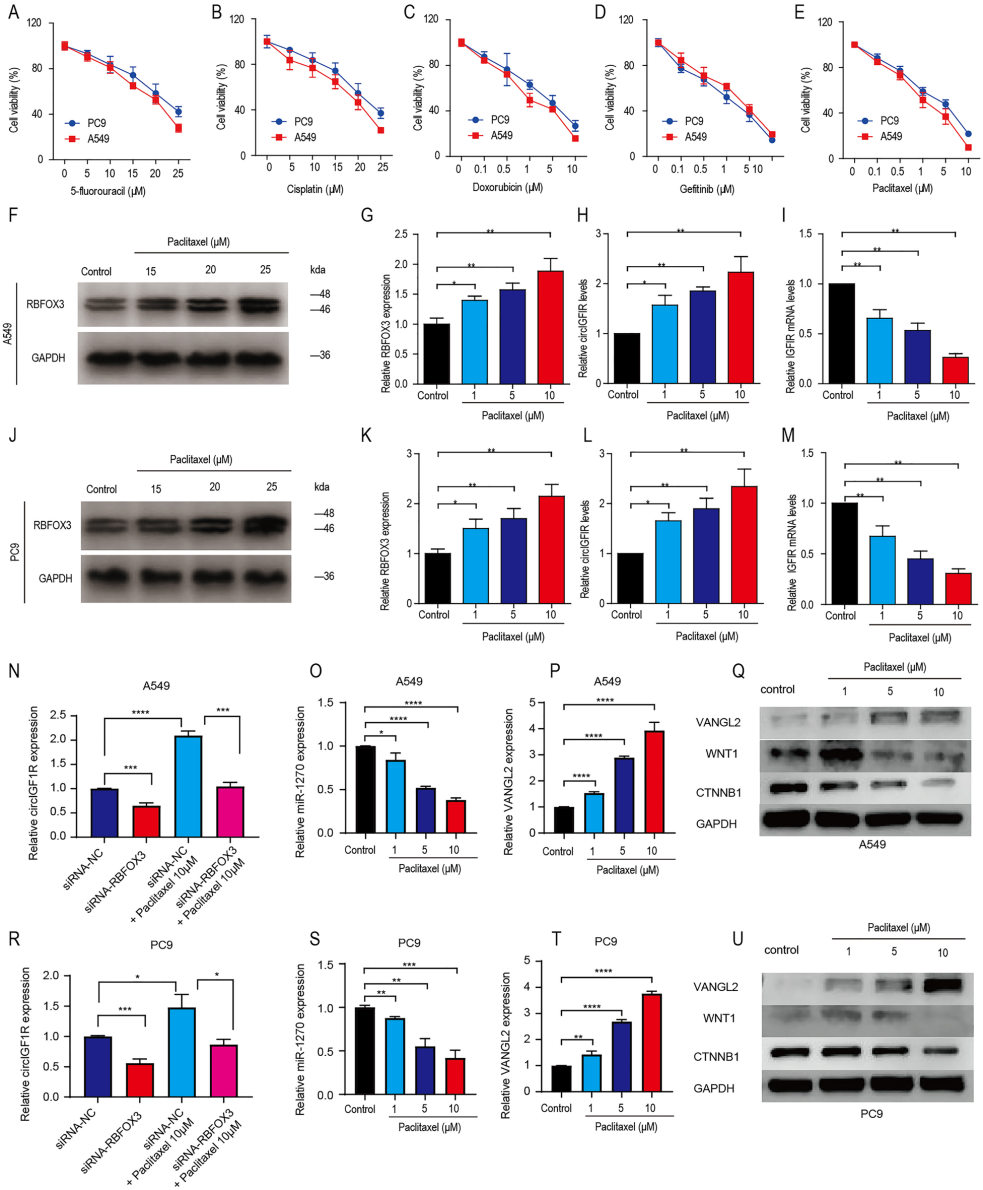
Paclitaxel affects the invasion and migration ability of A549 and PC9 cells. (**A–E**) Relative viability of A549 and PC9 cells after 24-h exposure to the indicated concentrations of 5-fluorouracil, cisplatin, paclitaxel, doxorubicin or gefitinib. (**F–M**) RBFOX3 protein, circIGF1R and IGF1R mRNA levels in A549 and PC9 cells treated with different concentrations of paclitaxel. (N, R) The relative expression levels of circIGF1R in siRNA-RBFOX3, Paclitaxel 10 μM, siRNA-RBFOX3 + Paclitaxel 10 μM transfected A549 and PC9 cells. (O-Q, S-U) Relative expression levels of miR-1270, VANGL2 and WNT pathway factors downstream of circRNA in paclitaxel-cultured A549 and PC9 cells. *p < 0.05, **p < 0.01, ***p < 0.001, ****p < 0.0001.

We compared the effects of different chemotherapeutic drugs on the viability of NSCLC cell lines by CCK-8 assay. As shown in Fig. 5A–E, 10 μM paclitaxel markedly reduced the viability of PC9 cells and A549 cells to 21.8% ± 0.49 and 9.73% ± 1.67 respectively, and its inhibitory effect was significantly greater (*p < 0.05) compared to that of 25 μM 5-fluorouracil (42.22% ± 4.38 and 27.83% ± 3.43), 25 μM cisplatin (37.16% ± 4.57 and 22.18% ± 1.34), 10 μM doxorubicin (21.8% ± 0.49 and 15.82% ± 1.34) and 10 μM gefitinib (14.4% ± 1.63 and 19.4% ± 1.44). Paclitaxel significantly increased the level of RBFOX3 protein (Fig. 5F–G,J–K) and circIGF1R (Fig. 5H,L), and downregulated IGF1R mRNA (Fig. 5I, M), whereas the other tested drugs had no significant effect on their expression levels (Supplementary Fig. S3). To investigate the RBFOX3-dependent role in circIGF1R biosynthesis, we found that circIGF1R expression decreased when siRNA-RBFOX3 was transfected in A549 and PC9 cells alone, increased when 10 μm Paclitaxel was cultured alone, and reversed when 10 μm Paclitaxel was cultured simultaneously with siRNA-RBFOX3 (Fig. 5N,R). As for the expression of circIGF1R downstream factors in A549 and PC9 cell lines under the effect of paclitaxel, the mRNA expression of miR-1270 decreased and that of VANGL2 increased, while the protein expression of VANGL2 increased and that of CTNNB1 decreased with the increase of Paclitaxel concentration, and the general trend of WNT1 expression decreased (Fig. 5O–Q,S–U).

**Figure 6:**
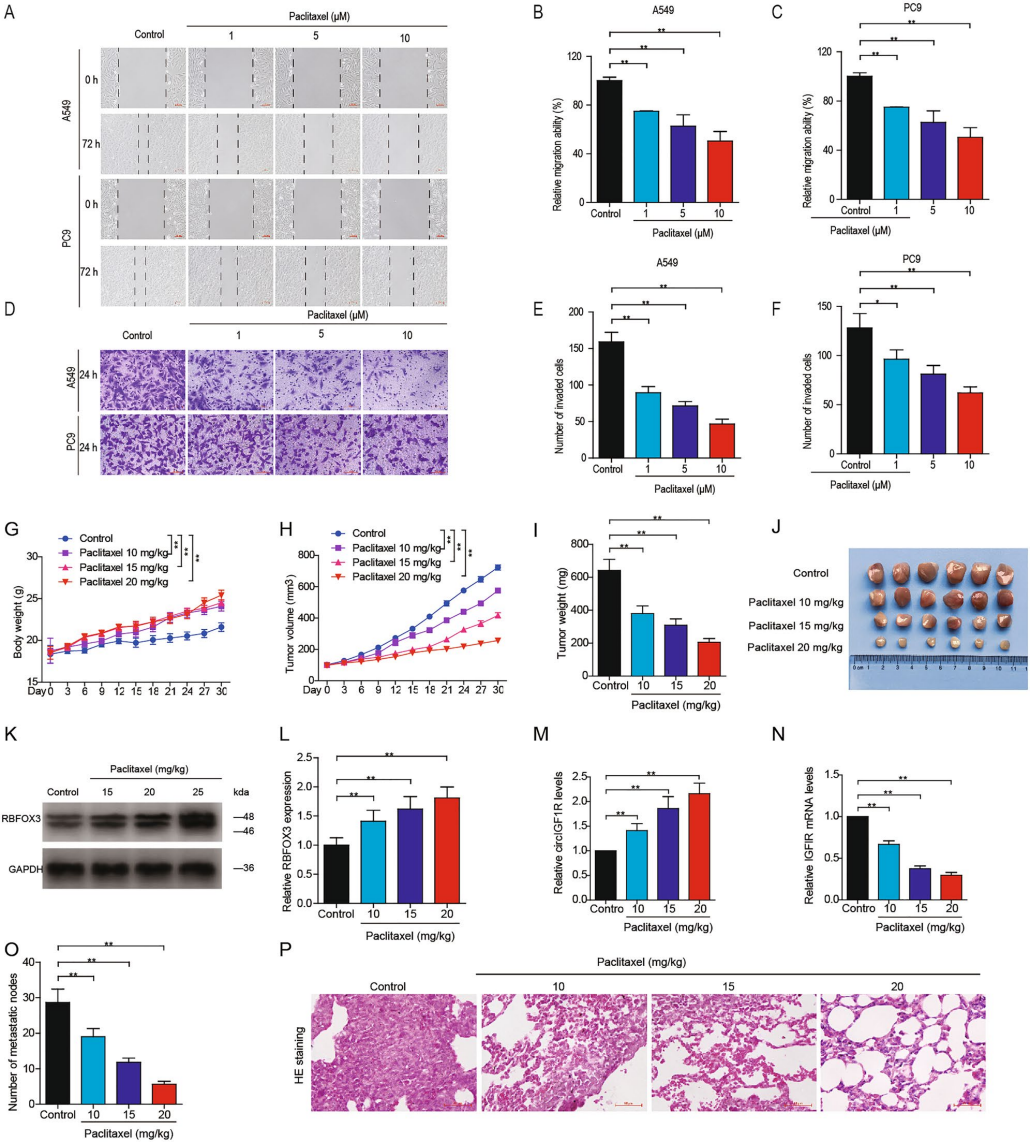
Effect of paclitaxel on NSCLC cells, PC9 xenograft growth and lung metastasis in nude mice. (**A**–**F**) Wound healing assay and Transwell migration assay to detect migration and invasion of paclitaxel-treated A549 and PC9 cells. (**G–J**) The Babl/c mouse were subcutaneously injected with 0.2 mL PC9 cells suspension (5 × 10^7^/mL) in the right axilla, followed by intraperitoneal injection of paclitaxel (10, 15 or 20 mg/kg i.p. once every 5 days). Body weight, tumor volume, tumor weight, and gross tumor images. (**K–N**) RBFOX3 protein, circIGF1R and IGF1R mRNA levels in the indicated groups. (O-P) PC9 cells were injected into the lateral tail vein of nude mice to induce lung metastasis, followed by intraperitoneal injections of paclitaxel (10, 15 or 20 mg/kg every 5 days). The number of metastatic nodes per lung and HE stained sections. **p < 0.01.

**Figure 7:**
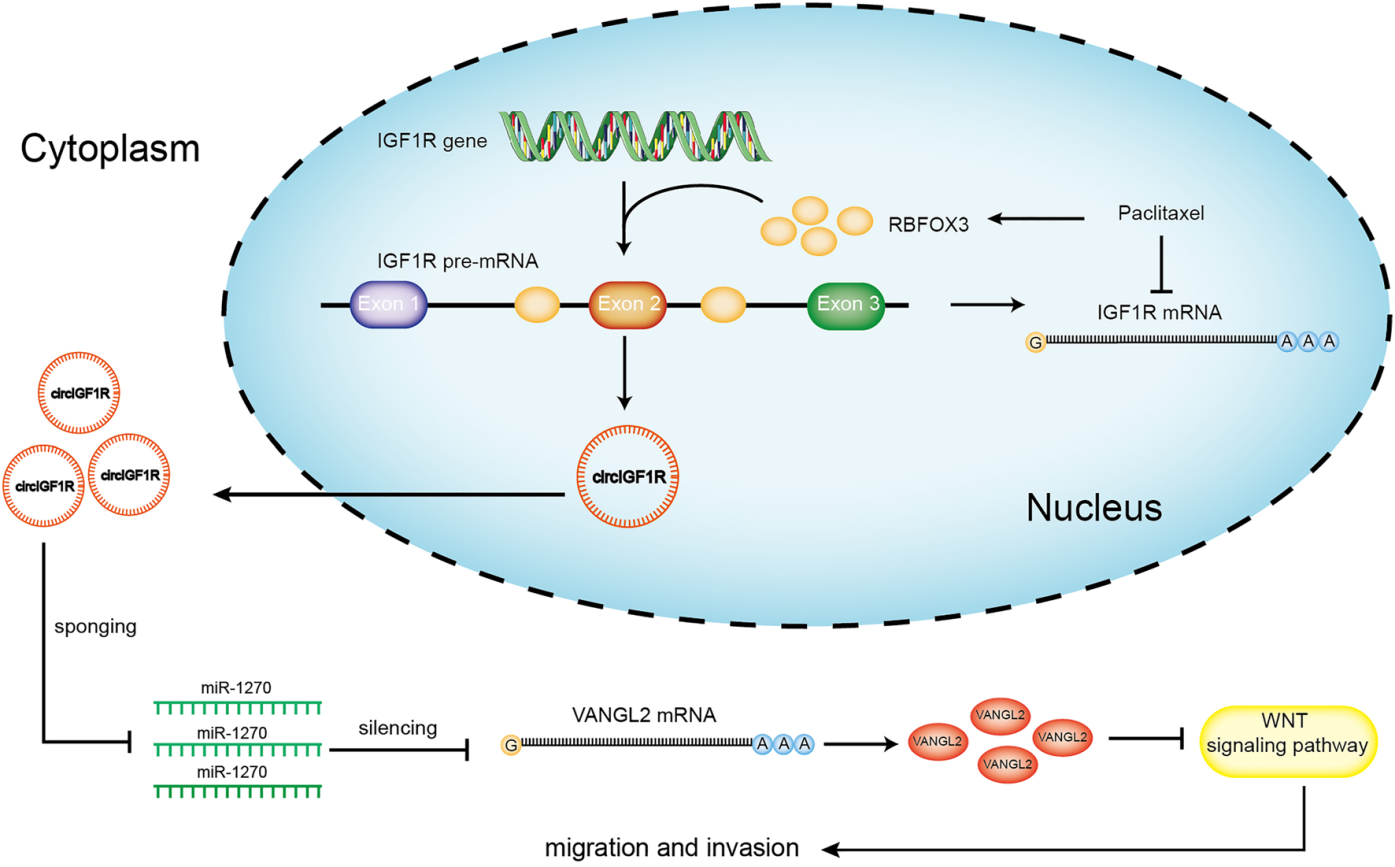
Schematic diagram demonstrating the inhibitory effect of circIGF1R on NSCLC migration and invasion via miR-1270/VANGL2/WNT pathway and that paclitaxel inhibits NSCLC invasion and migration by increasing circIGF1R biogenesis via RBFOX3.

As expected, incubation with paclitaxel led to a marked decrease in invasion and migration ability of A549 and PC9 cells in a dose-dependent manner (Fig. 6A–F).In the PC9 xenograft-bearing mice as well, paclitaxel accelerated body weight gain (Fig. 6G), slowed tumor growth (Fig. 6H), significantly reduced tumor mass (Fig. 6I, J). At the molecular level, paclitaxel upregulated the RBFOX3 protein (Fig. 6K,L) and circIGF1R (Fig. 6M) in the tumor tissues, and downregulated IGF1R mRNA (Fig. 6N). Furthermore, paclitaxel also led to a marked decrease in the number of PC9 lung metastatic nodules and increase in the body weight of the tumor-bearing mice (Fig. 6O–P). The inhibitory effects of paclitaxel were dose-dependent. Thus, paclitaxel inhibits NSCLC cell invasion and migration by inducing RBFOX3, which in turn increased the expression level of circIGF1R. Schematic diagram (Fig. 7) demonstrating the inhibitory effect of circIGF1R on NSCLC migration and invasion via miR-1270/VANGL2/WNT pathway and that paclitaxel inhibits NSCLC invasion and migration by increasing circIGF1R biogenesis via RBFOX3.

## Discussion

Studies show that circRNAs sponge miRNAs to weaken their inhibitory effect on the target mRNAs. In lung squamous cell carcinoma, circTP63 binds competitively to miR-873-3p and prevents it from suppressing FOXM1, thereby upregulating CENPA and CENPB and promoting cell cycle progression^16^. The chemosensitivity of triple negative breast cancer (TNBC) cells is regulated by the circWAC/miR-142/WWP1 ceRNA network via the PI3K/AKT pathway^17^. We found that circIGF1R and miR-1270 co-localized in the cytoplasm and interacted directly with each other. CircIGF1R overexpression blocked the invasion and migration of NSCLC cells, whereas its knockdown had the opposite effect, indicating that it functions as a tumor suppressor. The pro-malignant phenotype of the circIGF1R-knockdown cells was reversed by the miR-1270 inhibitor, and the mimic construct promoted the invasion and migration of NSCLC cells. Compared to previous studies, our findings reveal a unique mechanism of circIGF1R acting as a miRNA sponge in NSCLC, contrasting with Zhao Zhao et al.^18^. observation where miR-1270 promotes tumorigenesis in ovarian cancer. This highlights the diversity and specificity of circRNA functions across different cancer types. Wenbo Yuan et al. similarly reported that mi-1270 induced chemoresistance in bladder cancer cells by competing with Cdr1as^19^. Thus, circIGF1R acts as a miRNA sponge to inhibit NSCLC cell invasion and migration. VANGL2, a tissue patterning and polarity factor^20^, was predicted as a miR-1270 target gene. It is overexpressed in breast cancer cells, and predicts poor prognosis on account of increased migration, and the anchorage-dependent and -independent proliferation of the tumor cells^21^. In our study, VANGL2 knockdown in PC9 cell lines overexpressing circIGF1R reversed the inhibitory effects of the latter. This indicates that circIGF1R acts suppresses NSCLC cells via the circIGF1R /miR-1270/VANGL2 axis. Additionally, circIGF1R suppresses the downstream WNT signaling pathway by acting through the ceRNA axis. A variety of human malignancies have been linked to dysregulation of the Wnt/β-catenin signaling pathway^22–24^. Inhibition of the Wnt signaling pathway can suppress NSCLC cell proliferation and xenograft growth, diminish cell migration and invasion, and generate a more differentiated phenotype, all of which are essential in NSCLC^25–28^. CTNNB1 accumulates in the nuclei of tumor cells when the Wnt signaling pathway is activated, resulting in the loss of epithelial integrity and increased tumor invasion and metastasis^29,30^. CTNNB1 can also increase the breakdown of extracellular matrix by up-regulating the production of MMPs via signal transduction^31^. WNT1 is now well established in the regulation of tumor cell migration and epithelial-mesenchymal transition^32,33^. WNT1 is increased in premalignant lesions of breast cancer, downregulating E-cadherin junctions and encouraging metastasis, according to Linde et al.^34^. Wnt1 is secreted by M2-type tumor-associated macrophages, triggering a Wnt/β-catenin-regulated network involved in the migration and proliferation of invasive thyroid carcinoma cells, according to zhan et al.^35^.

We identified 87 circIGF1R-interacting proteins, of which the splicing factor RBFOX3 interacted with circIGF1R in PC9 cells. The Rbfox3 gene encodes the RNA-binding protein fox-1 homolog 3 (RBFOX3), which belongs to the fox1 splicing factor family^36^. RBFOX3 modulates various alternative pre-mRNA splicing events in the brain tissues by interacting with RNA penta (hexa) nucleotide (U) GCAUG motif^37^. Tianze Liu et al. showed that RBFOX3 binds the hTERT promoter in hepatocellular carcinoma cells, and accelerates cell growth and migration^38^. We found that RBFOX3 bound to the pre-mRNA of IGF1R and regulate the biogenesis of circIGF1R, and predicted their binding sites through bioinformatics. RIP assay showed that RBFOX3 bound strongly to the pre-mRNA flanking the near circIGF1R cyclization site. Furthermore, RBFOX3 produced significantly higher levels of circIGF1R from the WT type minigene compared to the MUT type. Overexpression of RBFOX3 in A549 and PC9 cells inhibited their migration and invasion, markedly elevated circIGF1R levels, and significantly reduced IGF1R mRNA. Yong-Eun Kim et al. reported a higher number of RBFOX3-positive cells in lung tumor tissues relative to normal lung tissues. In addition, TGF-β1 suppresses EMT of lung cancer cells by inducing RBFOX3^39^. Taken together, RBFOX3 inhibits the malignant phenotype of NSCLC cells by increasing circIGF1R biogenesis through binding with the IGF1R pre-mRNA, which inhibits IGF1R mRNA synthesis.

Paclitaxel has been used to treat patients with advanced lung cancer since 1992^40^. It induces cell cycle at the G2/M phase, thereby promoting cancer cell death^41^. At present, paclitaxel is also a promising chemotherapeutic agent for treating advanced NSCLC(43). Paclitaxel significantly inhibited the invasion and migration of the NSCLC cells in vitro and tumor growth in vivo in a dose-dependent manner. We have shown for the first time that paclitaxel upregulates circIGF1R in lung cancer cells by inducing RBFOX3 and downregulates IGF1R mRNA. These results suggest that paclitaxel inhibits NSCLC cells by inducing RBFOX3 and circIGF1R biogenesis. Our study may also provide a theoretical basis for the application of paclitaxel in advanced metastatic NSCLC.

Our research, substantiated by in vitro and animal model validations, highlights the need for clinical translation to assess circIGF1R's therapeutic efficacy in NSCLC. Future endeavors should prioritize translating these discoveries into clinical settings and probing circIGF1R's potential as a biomarker for NSCLC, especially in forecasting treatment outcomes and disease trajectory. Moreover, the critical role of RBFOX3 in circIGF1R biogenesis warrants further exploration, both to elucidate the underlying regulatory mechanisms and to pioneer novel therapeutic approaches leveraging this pathway.

In conclusion, circIGF1R acts a tumor suppressor in NSCLC by inhibiting the Wnt pathway through the VANGL2/miR-1270 axis, RBFOX3 promotes circIGF1R biogenesis by binding to IGF1R pre-mRNA, and is induced by paclitaxel to inhibit the migration and invasion of NSCLC cells. Thus, circIGF1R has potential to serve as a biomarker and therapeutic target for NSCLC.

## Methods

### Cell lines

Normal human fibroblast lung cells (MRC-5) and human NSCLC cell lines (A549, H1299, Calu-1, H1975, H1650) were purchased from the American Type Culture Collection (ATCC). PC9 cells were purchased from Chinese Academy of Sciences (Shanghai, China). The A549 cells were cultured in F-12 K medium (Gibco), H1650, H1975 and H1299 cells in RPMI-1640 medium (Gibco), MRC-5 cells in EMEM (Gibco), PC9 cells in DMEM (Gibco), and Calu-1 cells in McCoy’s 5A medium (Gibco). All culture media were supplemented with 10% FBS, and the cell lines were cultured at 37 °C under 5% CO_2_.

### Sample collection

This study was approved by the ethics committee of the First Affiliated Hospital of Guangxi Medical University (Approval No. 2016-KY-NSFC-098).We confirm that all experiments were performed in accordance with relevant guidelines and regulations.

Fifty paired NSCLC and non-cancerous lung tissue samples were obtained from the First Affiliated Hospital of Guangxi Medical University from February 2015 to September 2018, and stored at -80 °C. NSCLC diagnosis was confirmed by two independent pathologists. All patients provided written informed consent before sample collection.

### Cell transfection

A549 and PC9 cells were transfected with circIGF1R siRNA. circIGF1R overexpression plasmid was obtained from General Biosystems (Hefei, China). The full-length RBFOX3 cDNA sequence was cloned into the pcDNA3.1 vector (GenePharma, Shanghai, China). A lentivirus expressing human RBFOX3-shRNA was also generated. MiR-1270 inhibitors/mimics were obtained from RiboBio (Guangzhou, China). All transfections were performed using Lipofectamine 2000 (Invitrogen, Carlsbad, CA) according to the manufacturer’s protocol. The sequences of siRNAs and shRNAs in this study were listed in Supplementary Table S1.

### RNA extraction and qRT-PCR

Total RNA was extracted from the cell lines and lung tissues using TRIzol kit (Invitrogen, Carlsbad, CA, USA) according to the manufacturer’s instructions. The RT-PCR primers were obtained from Cellcook (Guangzhou, China) and are listed in Supplementary Table S2. QRT-PCR was conducted on the ABI7300 system (Applied Biosystems) using SYBR green kit (TaKaRa, Dalian, China) as per the manufacturer’s instructions. U6 or Glyceraldehyde 3-phosphate dehydrogenase (GAPDH) were used as endogenous controls. Comparative Ct method (2^-ΔΔCt^) was used to measure relative gene expression levels.

### Western blotting

Proteins were isolated using the RIPA lysis buffer (Geneseed Biotech, GuangZhou, China), separated by 10% SDS-PAGE and transferred to PVDF membranes. The blots were incubated overnight with primary antibodies, followed by the horseradish peroxidase (HRP)-labeled secondary antibody. The positive bands were detected using an enhanced chemiluminescence (ECL) substrate kit (Invitrogen). The antibodies are listed in Supplementary Table S3.

### Transwell migration and invasion assay

Corning Transwell® chambers were used for the invasion assay. A549 and PC9 cells were seeded on the chambers placed into 24-well plates and cultured for 48 h. The cells present on the lower surface of the chambers were fixed with 4% formaldehyde, stained with 0.25% crystal violet, and counted. The in vitro migration of these cells was evaluated by the wound-healing assay. Briefly, A549 and PC9 cells were seeded into the 6-well plates with 1 × 10^6^ cells per well (Corning) and cultured till 80%–90% confluence. A longitudinal scratch was made using a sterile pipette, and images were taken at 0, 24 and 48 h to monitor wound coverage.

### Dual luciferase reporter assay

The mutant (MUT) and wild type (WT) circIGF1R sequences were cloned into the pmir-GLO vector (Promega, USA), and transfected into A549 cells using Lipofectamine 2000 (Thermo Fisher, USA). Luciferase activity was quantified 48 h later. To validate the interplay between miR-1270 and VANGL2, the luciferase activities of MUT and VANGL2 3′UTR WT were similarly quantified.

### Fluorescence in situ hybridization (FISH)

The subcellular location of miR-1270 and circIGF1R was determined using the FISH assay. The FISH probe was prepared by GenePharma (Shanghai, China). In the fluorescent probes, FAM was served as a marker of miR-1270 whereas Cy3 served as a marker of circIGF1R. Cell nuclei were stained with DAPI dye. Results of the FISH assay were analyzed with confocal microscopy (Leica, Germany).

### Biotin-labelled RNA pull-down assay

For the circRNA-miRNA interaction, RNA pull-down assay was performed based on streptavidin magnetic beads (Life Technologies, CA, USA) and 3 biotin-labeled probes specific to circIGF1R back-splice region. Next, A549cells were lysed and cultured with magnetic beads coated with the circIGF1R-targeting biotin-coupled probe. Trizol reagent was used to separate RNAs from the pulled-down components. qPCR was used to detect the enrichment of circIGF1R and miR-1270.

In addition, Pierce Magnetic RNA–Protein Pull-Down Kit was used to conduct an RNA pull down test to investigate RNA–protein interaction (ThermoFisher Scientific). The RNA pulldown was carried out as directed by the kit. Here were the basic steps: In summary, the biotin-labelled RNA was first mixed with 75 μL streptavidin magnetic beads. About 8 × 107 A549 cells were treated with entire protein, while nucleic acids were eliminated from the extracted samples. We then mixed the cell extract and the probe-magnetic beads complex and culture them for 2 h at room temperature. Finally, the obtained RNA-binding proteins (RNPs) were characterized with the rapid silver staining kit (Beyotime, Shanghai, China). The Q Exactive mass spectrometer (Thermo Fisher) was utilized for RNPs detection which were examined in reference to the UniProt Homo sapiens database (https://www.uniprot.org).

### RNA immunoprecipitation (RIP)

RIP assay was performed using the Imprint RNA Immunoprecipitation Kit Geneseed Biotech, GuangZhou, China). A549 cells were lysed with the RIP lysis buffer, and equal amount of lysates were incubated with magnetic beads conjugated with anti-RBFOX3 or non-specific IgG (Abcam) at 4 °C. Total RNA was extracted from the precipitated RNA, and quantified by RT-qPCR.

### Minigene assay

The primers were designed based on the sequences of the exon region where circIGF1R is located and the intron region containing the RBFOX3 splice site bilaterally, and the primers were used to amplify the IGF1R gene Exon2 and the 5' end or 3' end of the bilaterally intron in the genome of A549 cells.The pcDNA3.1(-) vector was digested with restriction endonucleases XbaI, XhoI, EcoRI at 37 °C to obtain the linearized expression vector, and the target fragment obtained above was ligated into the linearized expression vector to construct WT-circRNA-minigene. The identified WT-circRNA-minigene plasmid was used as a template for reverse PCR, and the predicted binding site sequences of both intron regions were mutated to construct MUT-circRNA-minigene according to the instructions of QuickMutationTM Plus Gene Targeting Mutagenesis Kit (Beyotime, ShangHai, China). After A549 cells were treated with ov-RBFOX3 for 24 h, 2 μg of pcDNA3.1 with WT-circRNA-minigene or MUT-circRNA-minigene was transfected for 24 h and then total RNAs were extracted using an RNeasy mini kit (Qiagen).

### Animal experiments

Animal experiments were approved by the The Institutional Animal Care and Use Committee at Guangxi Medical University's First Affiliated Hospital, and were conducted according to relevant national and international guidelines. All animal methods are reported in accordance with ARRIVE guidelines. Six-week-old male nude mice (BALB/c) were provided by the Animal Center of Nanjing University (Nanjing, China). To induce subcutaneous tumors, the mice (10 per group) were each injected with 5 × 10^7^ PC9 cells in 0.2 ml into their limbs, these mice were then divided into three groups of six mice each, and paclitaxel was administered intraperitoneally at doses of 10, 15, and 20 mg/kg every five days, which was a reasonable dosing schedule based on relevant published studies, and its safety was confirmed by reported toxicity tests at all dose levels^42–45^. The tumors were measured every three days using calipers once they were palpable, and the tumor volume was calculated as (width^2^ × length)/2. The sample size for these tests was not predefined.

To establish the metastasis model, 1 × 10^5^ cells were injected into the tail veins of each mouse (6 per group), and lung metastases was tracked using the Xenogen IVIS Spectrum Imaging System (PerkinElmer, USA). The lungs and liver tissues were removed after 8 weeks, and the number of macroscopically evident metastatic nodules was counted. The nodules were then cut into thin sections and stained with hematoxylin and eosin (HE) for histopathological validation.

### Cell counting Kit-8 proliferation assay

A549 and PC9 cells were seeded into 96-well plates at the density of 3 × 10^3^/ml, and treated with the different compounds for 48 h. The serum was replaced with fresh serum-free medium containing 10 µl CCK-8 assay, and the cells were incubated further for 4 h. The optical density at 450 nm was measured using a Multiskan FC (Thermo Fisher Scientific, USA).

### Hematoxylin and eosin (HE) staining

Excised mice lung tissues were fixed with formaldehyde, embedded in paraffin, and cut into thin sections. Hematoxylin and eosin staining was performed for 9 and 4 min respectively, and the stained sections were examined under a microscope (Nikon, Japan).

### Statistical analysis

Statistical analyses were conducted using SPSS (versions 22.0) and GraphPad Prism (versions 8.0). Continuous data were evaluated using paired and unpaired Student’s *t*-tests, while categorical data were analyzed with the χ2 test. Differences between groups were assessed using. All tests were two-sided, with a *p*-value of < 0.05 indicating statistical significance.

### Ethics approval

The Institutional Animal Care and Use Committee at Guangxi Medical University's First Affiliated Hospital authorized all animal trials. The study adhered to the National Institutes of Health's Guide for the Care and Use of Laboratory Animals (National Research Council).

### Supplementary Information

Supplementary Information.

## Data Availability

All data needed to evaluate the conclusions in the paper are present in the paper and/or the Supplemental Information. Additional data are available from Zhanyu Xu upon request.
