# Supplementary material for: Paclitaxel-induced inhibition of NSCLC invasion and migration via RBFOX3-mediated circIGF1R biogenesis
Source: Sci Rep. 2024 Jan 8;14:774. doi: 10.1038/s41598-024-51500-1 (PMC10774373; doi:10.1038/s41598-024-51500-1)
Supplement: Supplementary file 1 — Supplementary Information. [file 41598_2024_51500_MOESM1_ESM.pdf]

Supplementary Information

Table S1 Sequences of siRNAs and shRNAs used in this study

**Definition**	**sequences**
si-circIGF1R	5’-ATGCCTGGCCCGCAGATTTTC-3’
si-NC	5’-GAAAATCTGCGGG CCAGGCAT-3’
sh-RBFOX3	5’CCGGGTCGTGTATCAGGATGGATTTCTCGAGAAA TCCATCCTGATACACGACTTTTTG-3'
sh-NC	5’CCGGCAACAAGATGAAGAGCACCAACTCGAGTT GGTGCTCTTCATCTTGTTGTTTTTG -3’

Table S2 Primer sequences used in RT-qPCR and PCR analysis

Gene	Primer Sequence (5’→3’)	
circIGF1R	Forward	CAAACCGCTGCCAGAAAATCT
	Reverse	GCTGCTGATAGTCGTTGCGGA
IGF1R	Forward	TCGACATCCGCAACGACTATC
	Reverse	CCAGGGCGTAGTTGTAGAAGAG
YBX1	Forward	CTGGTTTTTCTCAATACGCC
	Reverse	TATTAACAGGTGCTTGCAGT
HNRNPM	Forward	AGAAAAAGGGATGTGCTGTT
	Reverse	CATGTTCACCATCAGGATCT
HNRNPH1	Forward	TGGAAGAGACCTCAATTACTG
	Reverse	GTTGTGCTCTGGAAAGTAGA
HNRNPF	Forward	TCCTCAAGGTTGAGAACAAA
	Reverse	TAATGAAATGTCCACGGAGG
RBFOX3	Forward	GAAATGTTCTTGGCTGTAGG
	Reverse	CACAGGAAGACTGTTAGCTT
FUS	Forward	ACATGGCCTCAAACGATTAT
	Reverse	GCTCTGGCCATAAGAAGAAT
GAPDH	Reverse	GCACCGTCAAGGCTGAGAAC
	Reverse	TGGTGAAGACGCCAGTGGA
a (RIP assay)	Forward	CGTGATGAGTCACTCCTATGA
	Reverse	TGGCACACTGGCCTTCAATTC
b (RIP assay)	Forward	CGGTTCTCCAGTGTAGAGTAG
	Reverse	CAGTGCCAACACTGGACCAGA
c (RIP assay)	Forward	CAAACCGCTGCCAGAAAATCT
	Reverse	GCTGCTGATAGTCGTTGCGGA
d (RIP assay)	Forward	CTTCTCTTCTGAGTGCACGAG
	Reverse	GAAGGTCAGCAAGTGTCTAGC
e (RIP assay)	Forward	TCTCTCTCTACCATTGCTGTC
	Reverse	GGCACCTTTAAGATTCACTGC

Table S3 Antibodies and dilution

Antibodies	Dilution (application)
RBFOX3	1:1000 (WB)
VANGL2	1:2000 (WB)
WNT1	1:1000 (WB)
CTNNB1	1:1000 (WB)
GAPDH	1:10000 (WB)
Goat Anti-Rabbit IgG H&L(HRP)	1:10000 (WB)
Rabbit Anti-Mouse IgG H&L(HRP)	1:10000 (WB)

Table S4 Mass Spectrometry Experiment Protein List

Protein number	Protein access number	Protein Descriptions
1	sp|P67809|YBOX1_HUMAN	Y-box-binding protein 1 OS=Homo sapiens OX=9606 GN=YBX1 PE=1 SV=3
2	sp|P02768|ALBU_HUMAN	Serum albumin OS=Homo sapiens OX=9606 GN=ALB PE=1 SV=2
3	sp|P13645|K1C10_HUMAN	Keratin, type I cytoskeletal 10 OS=Homo sapiens OX=9606 GN=KRT10 PE=1 SV=6
4	sp|P04264|K2C1_HUMAN	Keratin, type II cytoskeletal 1 OS=Homo sapiens OX=9606 GN=KRT1 PE=1 SV=6
5	sp|P35908|K22E_HUMAN	Keratin, type II cytoskeletal 2 epidermal OS=Homo sapiens OX=9606 GN=KRT2 PE=1 SV=2
6	sp|P60709|ACTB_HUMAN	Actin, cytoplasmic 1 OS=Homo sapiens OX=9606 GN=ACTB PE=1 SV=1
7	sp|P35579|MYH9_HUMAN	Myosin-9 OS=Homo sapiens OX=9606 GN=MYH9 PE=1 SV=4
8	sp|P08670|VIME_HUMAN	Vimentin OS=Homo sapiens OX=9606 GN=VIM PE=1 SV=4
9	sp|P62753|RS6_HUMAN	40S ribosomal protein S6 OS=Homo sapiens OX=9606 GN=RPS6 PE=1 SV=1
10	sp|P52272|HNRPM_HUMAN	Heterogeneous nuclear ribonucleoprotein M OS=Homo sapiens OX=9606 GN=HNRNPM PE=1 SV=3
11	sp|P15927|RFA2_HUMAN	Replication protein A 32 kDa subunit OS=Homo sapiens OX=9606 GN=RPA2 PE=1 SV=1
12	sp|P31943|HNRH1_HUMAN	Heterogeneous nuclear ribonucleoprotein H OS=Homo sapiens OX=9606 GN=HNRNPH1 PE=1 SV=4
13	sp|P13647|K2C5_HUMAN	Keratin, type II cytoskeletal 5 OS=Homo sapiens OX=9606 GN=KRT5 PE=1 SV=3
14	sp|P52597|HNRPF_HUMAN	Heterogeneous nuclear ribonucleoprotein F OS=Homo sapiens OX=9606 GN=HNRNPF PE=1 SV=3
15	sp|P25705|ATPA_HUMAN	ATP synthase subunit alpha, mitochondrial OS=Homo sapiens OX=9606 GN=ATP5F1A PE=1 SV=1

<table>
<tr><td>16</td><td>sp|P05787|K2C8_HUMAN</td><td>Keratin, type II cytoskeletal 8 OS=Homo sapiens OX=9606 GN=KRT8 PE=1 SV=7</td></tr>
<tr><td>17</td><td>sp|P16989|YBOX3_HUMAN</td><td>Y-box-binding protein 3 OS=Homo sapiens OX=9606 GN=YBX3 PE=1 SV=4</td></tr>
<tr><td>18</td><td>sp|Q9NSU2|TREX1_HUMAN</td><td>Three-prime repair exonuclease 1 OS=Homo sapiens OX=9606 GN=TREX1 PE=1 SV=2</td></tr>
<tr><td>19</td><td>sp|P02533|K1C14_HUMAN</td><td>Keratin, type I cytoskeletal 14 OS=Homo sapiens OX=9606 GN=KRT14 PE=1 SV=4</td></tr>
<tr><td>20</td><td>sp|P07437|TBB5_HUMAN</td><td>Tubulin beta chain OS=Homo sapiens OX=9606 GN=TUBB PE=1 SV=2</td></tr>
<tr><td>21</td><td>sp|Q9UL12|SARDH_HUMAN</td><td>Sarcosine dehydrogenase, mitochondrial OS=Homo sapiens OX=9606 GN=SARDH PE=1 SV=1</td></tr>
<tr><td>22</td><td>sp|P21333|FLNA_HUMAN</td><td>Filamin-A OS=Homo sapiens OX=9606 GN=FLNA PE=1 SV=4</td></tr>
<tr><td>23</td><td>sp|O43795|MYO1B_HUMAN</td><td>Unconventional myosin-Ib OS=Homo sapiens OX=9606 GN=MYO1B PE=1 SV=3</td></tr>
<tr><td>24</td><td>sp|Q5VZL5|ZMYM4_HUMAN</td><td>Zinc finger MYM-type protein 4 OS=Homo sapiens OX=9606 GN=ZMYM4 PE=1 SV=1</td></tr>
<tr><td>25</td><td>sp|Q765P7|MTSS2_HUMAN</td><td>Protein MTSS 2 OS=Homo sapiens OX=9606 GN=MTSS2 PE=1 SV=1</td></tr>
<tr><td>26</td><td>sp|Q9Y6N5|SQOR_HUMAN</td><td>Sulfide:quinone oxidoreductase, mitochondrial OS=Homo sapiens OX=9606 GN=SQOR PE=1 SV=1</td></tr>
<tr><td>27</td><td>sp|A3KN83|SBNO1_HUMAN</td><td>Protein strawberry notch homolog 1 OS=Homo sapiens OX=9606 GN=SBNO1 PE=1 SV=1</td></tr>
<tr><td>28</td><td>sp|Q13126|MTAP_HUMAN</td><td>S-methyl-5~-thioadenosine phosphorylase OS=Homo sapiens OX=9606 GN=MTAP PE=1 SV=2</td></tr>
<tr><td>29</td><td>sp|P0DMR3|AT8OS_HUMAN</td><td>Putative protein ATXN8OS OS=Homo sapiens OX=9606 GN=ATXN8OS PE=5 SV=1</td></tr>
<tr><td>30</td><td>sp|Q2NKJ3|CTC1_HUMAN</td><td>CST complex subunit CTC1 OS=Homo sapiens OX=9606 GN=CTC1 PE=1 SV=2</td></tr>
<tr><td>31</td><td>sp|Q5R372|RBG1L_HUMAN</td><td>Rab GTPase-activating protein 1-like OS=Homo sapiens OX=9606 GN=RABGAP1L PE=1 SV=1</td></tr>
<tr><td>32</td><td>sp|Q00536|CDK16_HUMAN</td><td>Cyclin-dependent kinase 16 OS=Homo sapiens OX=9606 GN=CDK16 PE=1 SV=1</td></tr>
<tr><td>33</td><td>sp|Q9BQI9|NRIP2_HUMAN</td><td>Nuclear receptor-interacting protein 2 OS=Homo sapiens OX=9606 GN=NRIP2 PE=1 SV=3</td></tr>
<tr><td>34</td><td>sp|P20700|LMNB1_HUMAN</td><td>Lamin-B1 OS=Homo sapiens OX=9606 GN=LMNB1 PE=1 SV=2</td></tr>
<tr><td>35</td><td>sp|A6NNC1|P12LL_HUMAN</td><td>Putative POM121-like protein 1-like OS=Homo sapiens OX=9606 PE=5 SV=3</td></tr>
<tr><td>36</td><td>sp|Q14527|HLTF_HUMAN</td><td>Helicase-like transcription factor OS=Homo sapiens OX=9606 GN=HLTF PE=1 SV=2</td></tr>
<tr><td>37</td><td>sp|Q15583|TGIF1_HUMAN</td><td>Homeobox protein TGIF1 OS=Homo sapiens OX=9606 GN=TGIF1 PE=1 SV=3</td></tr>
</table>

38	sp|P08779|K1C16_HUMAN	Keratin, type I cytoskeletal 16 OS=Homo sapiens OX=9606 GN=KRT16 PE=1 SV=4
39	sp|P27144|KAD4_HUMAN	Adenylate kinase 4, mitochondrial OS=Homo sapiens OX=9606 GN=AK4 PE=1 SV=1
40	sp|Q9NZU0|FLRT3_HUMAN	Leucine-rich repeat transmembrane protein FLRT3 OS=Homo sapiens OX=9606 GN=FLRT3 PE=1 SV=1
41	sp|Q2UY09|COSA1_HUMAN	Collagen alpha-1(XXVIII) chain OS=Homo sapiens OX=9606 GN=COL28A1 PE=2 SV=2
42	sp|Q5H9R4|ARMX4_HUMAN	Armadillo repeat-containing X-linked protein 4 OS=Homo sapiens OX=9606 GN=ARMCX4 PE=2 SV=3
43	sp|Q2WGJ9|FR1L6_HUMAN	Fer-1-like protein 6 OS=Homo sapiens OX=9606 GN=FER1L6 PE=2 SV=2
44	sp|P36406|TRI23_HUMAN	E3 ubiquitin-protein ligase TRIM23 OS=Homo sapiens OX=9606 GN=TRIM23 PE=1 SV=1
45	sp|Q8WY64|MYLIP_HUMAN	E3 ubiquitin-protein ligase MYLIP OS=Homo sapiens OX=9606 GN=MYLIP PE=1 SV=2
46	sp|Q96I15|SCLY_HUMAN	Selenocysteine lyase OS=Homo sapiens OX=9606 GN=SCLY PE=1 SV=4
47	sp|Q10588|BST1_HUMAN	ADP-ribosyl cyclase/cyclic ADP-ribose hydrolase 2 OS=Homo sapiens OX=9606 GN=BST1 PE=1 SV=2
48	sp|O75764|TCEA3_HUMAN	Transcription elongation factor A protein 3 OS=Homo sapiens OX=9606 GN=TCEA3 PE=1 SV=2
49	sp|P27694|RFA1_HUMAN	Replication protein A 70 kDa DNA-binding subunit OS=Homo sapiens OX=9606 GN=RPA1 PE=1 SV=2
50	sp|Q9H1I8|ASCC2_HUMAN	Activating signal cointegrator 1 complex subunit 2 OS=Homo sapiens OX=9606 GN=ASCC2 PE=1 SV=3
51	sp|Q96DU9|PABP5_HUMAN	Polyadenylate-binding protein 5 OS=Homo sapiens OX=9606 GN=PABPC5 PE=2 SV=1
52	sp|Q9BRU9|UTP23_HUMAN	rRNA-processing protein UTP23 homolog OS=Homo sapiens OX=9606 GN=UTP23 PE=1 SV=2
53	sp|Q14894|CRYM_HUMAN	Ketimine reductase mu-crystallin OS=Homo sapiens OX=9606 GN=CRYM PE=1 SV=1
54	sp|Q8WXD9|CSKI1_HUMAN	Caskin-1 OS=Homo sapiens OX=9606 GN=CASKIN1 PE=1 SV=1
55	sp|P0C874|S31D3_HUMAN	Spermatogenesis-associated protein 31D3 OS=Homo sapiens OX=9606 GN=SPATA31D3 PE=2 SV=1
56	sp|Q13368|MPP3_HUMAN	MAGUK p55 subfamily member 3 OS=Homo sapiens OX=9606 GN=MPP3 PE=1 SV=2
57	sp|P26374|RAE2_HUMAN	Rab proteins geranylgeranyltransferase component A 2 OS=Homo sapiens OX=9606 GN=CHML PE=1 SV=2
58	sp|Q7Z5P9|MUC19_HUMAN	Mucin-19 OS=Homo sapiens OX=9606 GN=MUC19 PE=1 SV=3
59	sp|P30414|NKT	NK-tumor recognition protein OS=Homo sapiens

	R_HUMAN	OX=9606 GN=NKTR PE=1 SV=2
60	sp|P27816|MAP4_HUMAN	Microtubule-associated protein 4 OS=Homo sapiens OX=9606 GN=MAP4 PE=1 SV=3
61	sp|A8MTQ0|NOTO_HUMAN	Homeobox protein notochord OS=Homo sapiens OX=9606 GN=NOTO PE=2 SV=2
62	sp|Q9NQL2|RRAGD_HUMAN	Ras-related GTP-binding protein D OS=Homo sapiens OX=9606 GN=RRAGD PE=1 SV=1
63	sp|Q7Z6B7|SRGP1_HUMAN	SLIT-ROBO Rho GTPase-activating protein 1 OS=Homo sapiens OX=9606 GN=SRGAP1 PE=1 SV=1
64	sp|Q14166|TTL12_HUMAN	Tubulin--tyrosine ligase-like protein 12 OS=Homo sapiens OX=9606 GN=TTLL12 PE=1 SV=2
65	sp|Q99518|FMO2_HUMAN	Dimethylaniline monooxygenase [N-oxide-forming] 2 OS=Homo sapiens OX=9606 GN=FMO2 PE=1 SV=5
66	sp|O94986|CE152_HUMAN	Centrosomal protein of 152 kDa OS=Homo sapiens OX=9606 GN=CEP152 PE=1 SV=4
67	sp|O14734|ACOT8_HUMAN	Acyl-coenzyme A thioesterase 8 OS=Homo sapiens OX=9606 GN=ACOT8 PE=1 SV=1
68	sp|Q9HCM7|FBSL_HUMAN	Fibrosin-1-like protein OS=Homo sapiens OX=9606 GN=FBRSL1 PE=1 SV=4
69	sp|Q9NQ79|CRAC1_HUMAN	Cartilage acidic protein 1 OS=Homo sapiens OX=9606 GN=CRTAC1 PE=1 SV=2
70	sp|O15379|HDAC3_HUMAN	Histone deacetylase 3 OS=Homo sapiens OX=9606 GN=HDAC3 PE=1 SV=2
71	sp|Q9ULN7|PNM8B_HUMAN	Paraneoplastic antigen-like protein 8B OS=Homo sapiens OX=9606 GN=PNMA8B PE=2 SV=4
72	sp|Q16798|MAON_HUMAN	NADP-dependent malic enzyme, mitochondrial OS=Homo sapiens OX=9606 GN=ME3 PE=2 SV=2
73	sp|Q0VG73|YC023_HUMAN	Putative uncharacterized protein LOC152225 OS=Homo sapiens OX=9606 PE=5 SV=1
74	sp|Q8WVM7|STAG1_HUMAN	Cohesin subunit SA-1 OS=Homo sapiens OX=9606 GN=STAG1 PE=1 SV=3
75	sp|A6NFN3|RFOX3_HUMAN	RNA binding protein fox-1 homolog 3 OS=Homo sapiens OX=9606 GN=RBFOX3 PE=2 SV=4
76	sp|Q14865|ARI5B_HUMAN	AT-rich interactive domain-containing protein 5B OS=Homo sapiens OX=9606 GN=ARID5B PE=1 SV=3
77	sp|Q9BWT6|MND1_HUMAN	Meiotic nuclear division protein 1 homolog OS=Homo sapiens OX=9606 GN=MND1 PE=1 SV=1
78	sp|Q2TAM9|TUSC1_HUMAN	Tumor suppressor candidate gene 1 protein OS=Homo sapiens OX=9606 GN=TUSC1 PE=1 SV=3
79	sp|P08922|ROS1_HUMAN	Proto-oncogene tyrosine-protein kinase ROS OS=Homo sapiens OX=9606 GN=ROS1 PE=1 SV=3
80	sp|P05120|PAI2_HUMAN	Plasminogen activator inhibitor 2 OS=Homo sapiens OX=9606 GN=SERPINB2 PE=1 SV=2

81	sp|P19883|FST_HUMAN	Follistatin OS=Homo sapiens OX=9606 GN=FST PE=1 SV=2
82	sp|P49895|IOD1_HUMAN	Type I iodothyronine deiodinase OS=Homo sapiens OX=9606 GN=DIO1 PE=2 SV=3
83	sp|Q04721|NOTC2_HUMAN	Neurogenic locus notch homolog protein 2 OS=Homo sapiens OX=9606 GN=NOTCH2 PE=1 SV=3
84	sp|P13569|CFTR_HUMAN	Cystic fibrosis transmembrane conductance regulator OS=Homo sapiens OX=9606 GN=CFTR PE=1 SV=3
85	sp|Q5T1A1|DCST2_HUMAN	DC-STAMP domain-containing protein 2 OS=Homo sapiens OX=9606 GN=DCST2 PE=2 SV=2
86	sp|Q15784|NDF2_HUMAN	Neurogenic differentiation factor 2 OS=Homo sapiens OX=9606 GN=NEUROD2 PE=1 SV=2
87	sp|Q9UL68|MYT1L_HUMAN	Myelin transcription factor 1-like protein OS=Homo sapiens OX=9606 GN=MYT1L PE=2 SV=3

Figure S1. circIGF1R binding protein and expression levels. (a) Metascape analysis of functional categories enriched in the circIGF1R binding proteins. (b-f) The relative expression levels of YBX1, FUS, HNRNPH1, HNRNPF and HNRNPM mRNAs in NSCLC and lung normal cell lines. (g-h) HNRNPH1 and HNRNPF mRNA levels in 15 paired NSCLC and para-cancerous tissues.

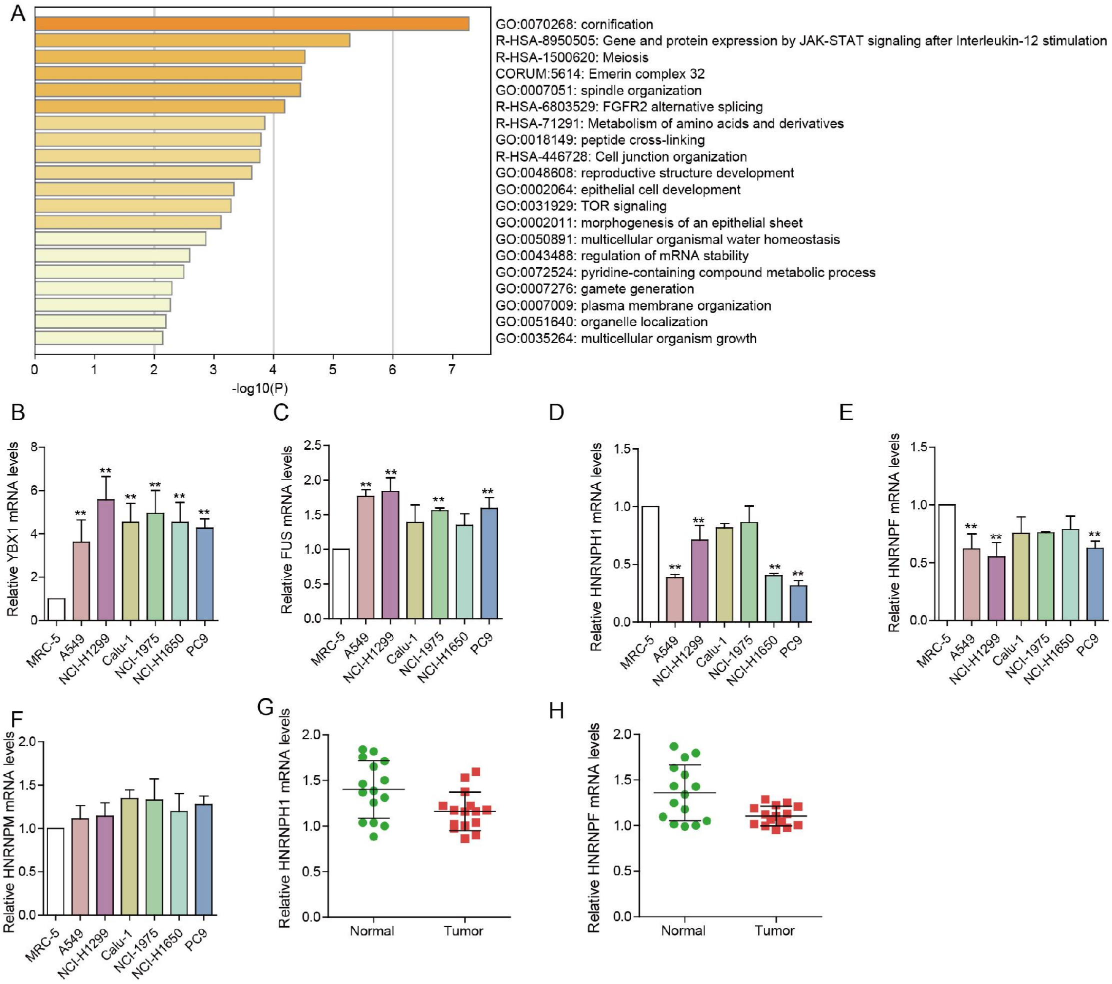

Figure S2. Analysis of transfection efficiency. (a-f) the protein and mRNA levels of RBFOX3 were significantly overexpressed in A549 and PC9 cells following transfection with the ov-RBFOX3 overexpression plasmid. (g-l) After transfection of A549 and PC9 cells with sh-RBFOX3 interference plasmid, the protein and mRNA levels of RBFOX3 were significantly low expressed.

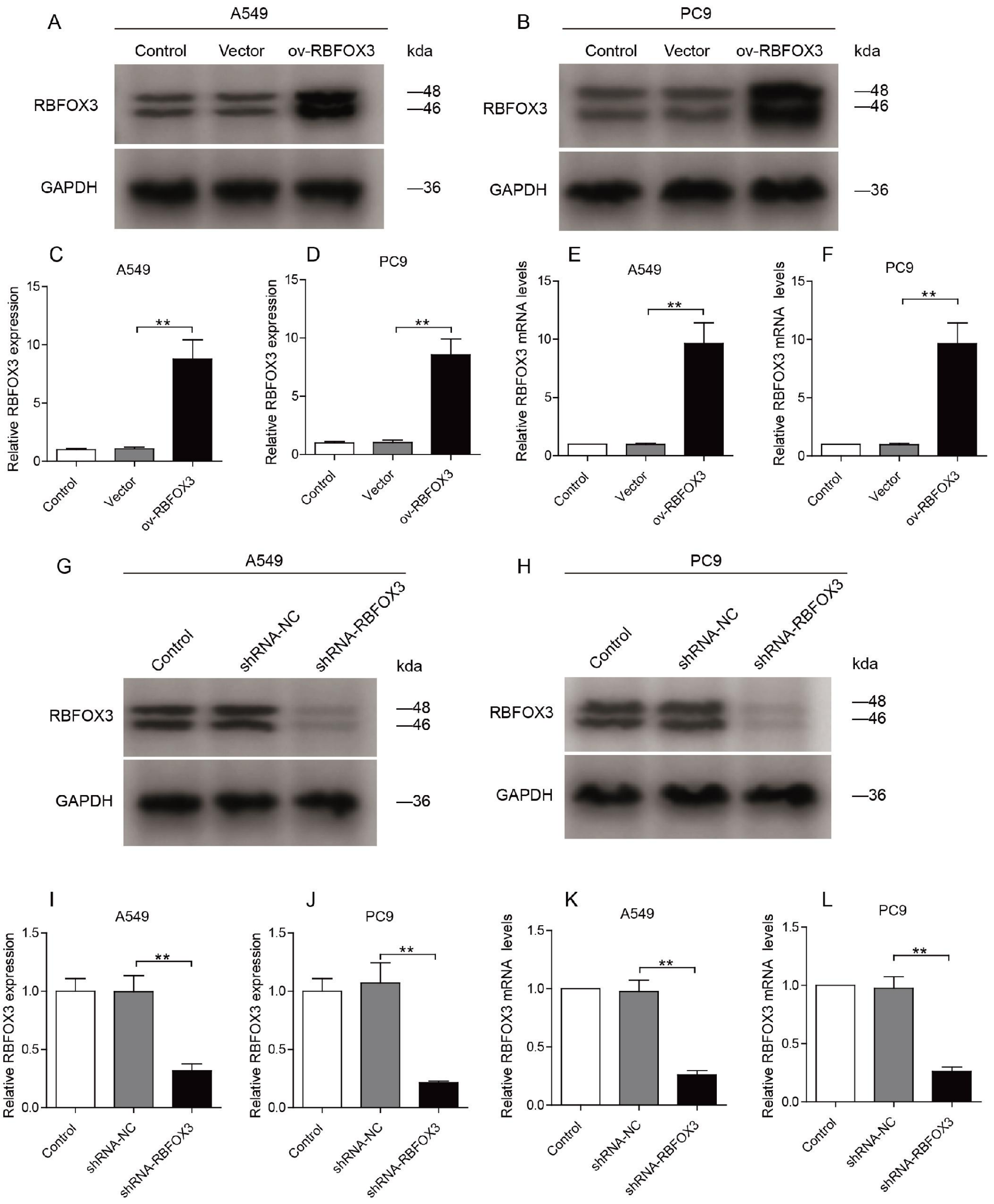

Figure S3. Effect of different drug treatments on A549 and PC9 cells. (a-h) RBFOX3 protein, IGF1R and circIGF1R mRNA levels in A549 and PC9 cells treated with different concentrations of 5-fluorouracil, cisplatin, doxorubicin, gefitinib.

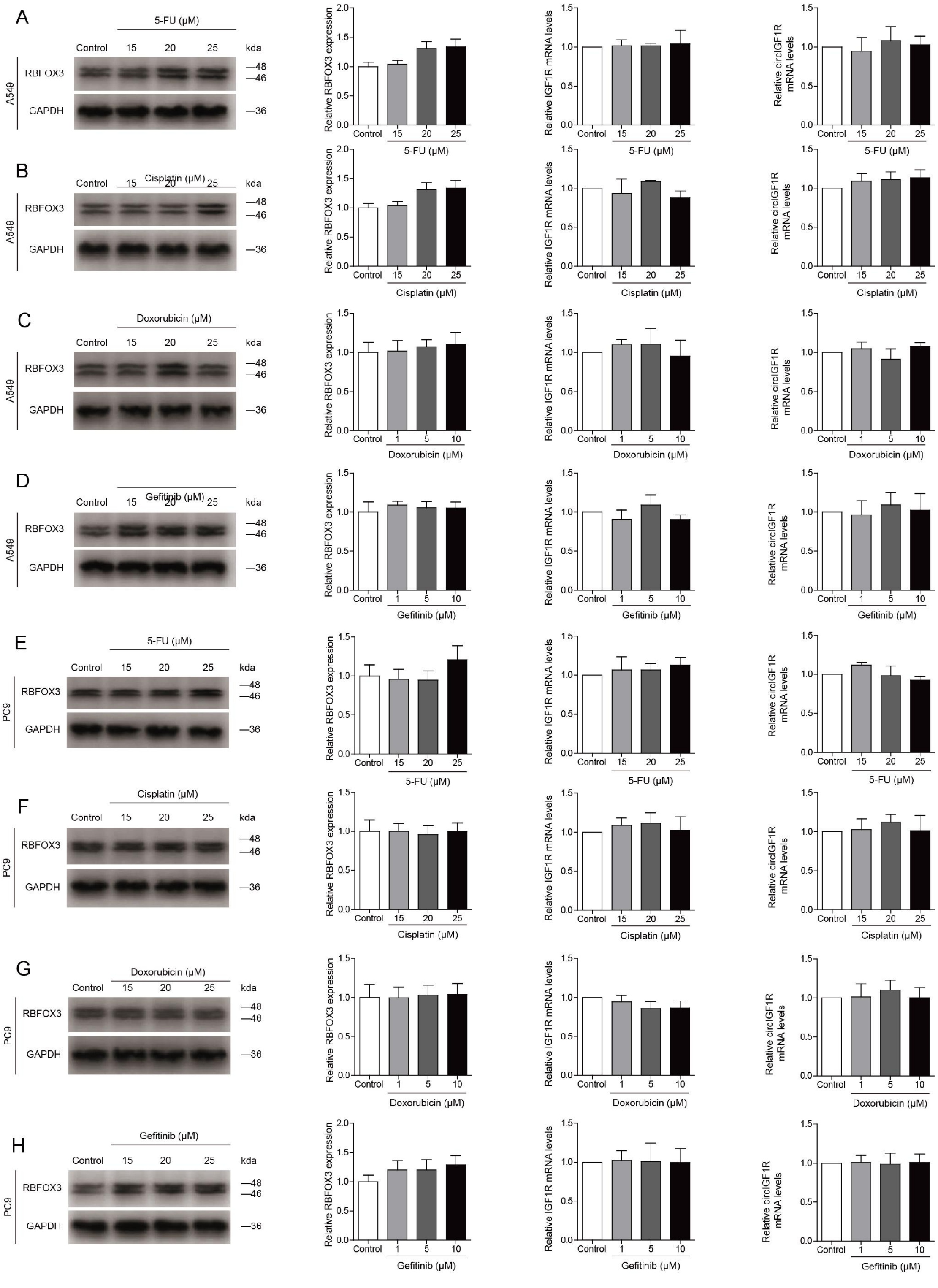

A
5-FU (μM)
Control 15 20 25 kda
RBFOX3
—48
—46
GAPDH
—36
A549
Relative RBFOX3 expression
Relative IGF1R mRNA levels
Relative circIGF1R mRNA levels
B
Cisplatin (μM)
C
Doxorubicin (μM)
Control 1 5 10
D
Gefitinib (μM)
E
PC9
F
G
H

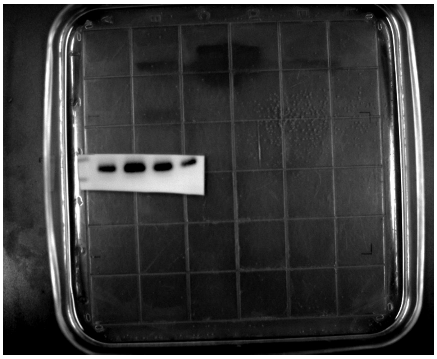

Fig 2i-CTNNB1

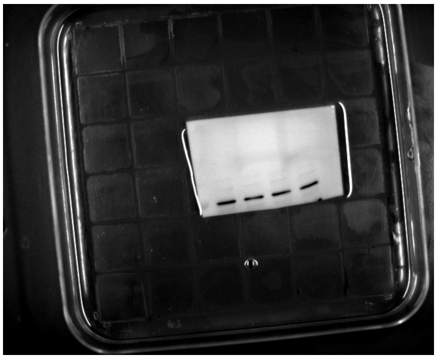

2i-GAPDH

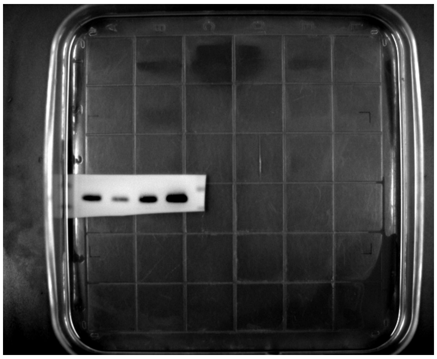

Fig 2i-VANGL2

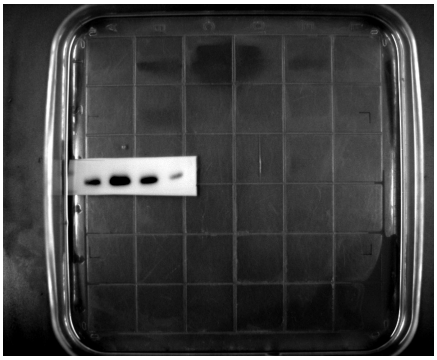

Fig 2i-WNT1

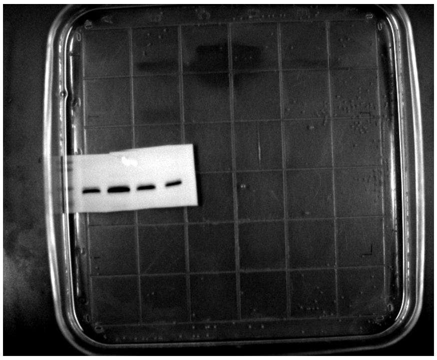

Fig 2m-CTNNB1

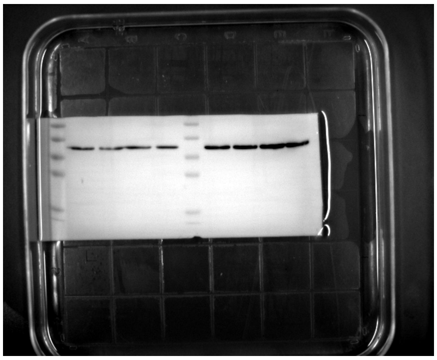

Fig 2m-GAPDH

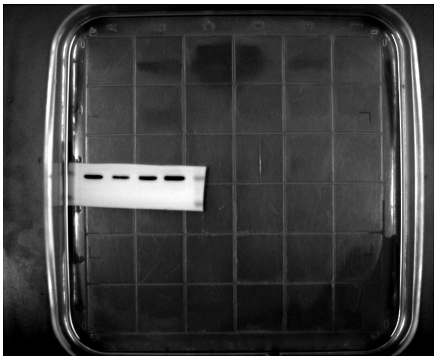

Fig 2m-VANGL2

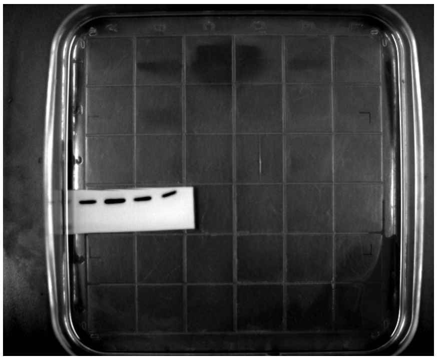

Fig 2m-WNT1

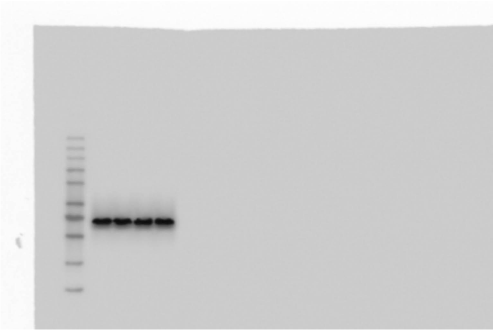

Fig 5f-GAPDH

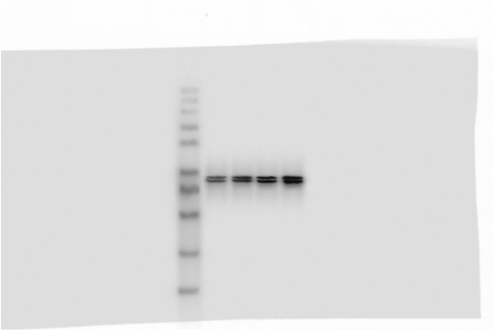

Fig 5f-RBFOX3

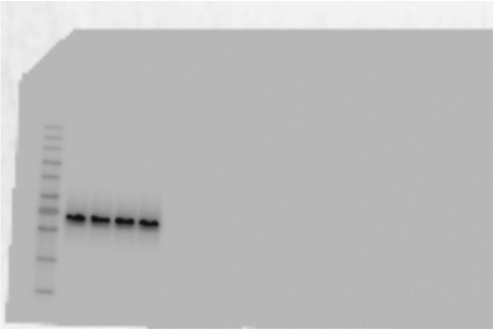

5j-GAPDH

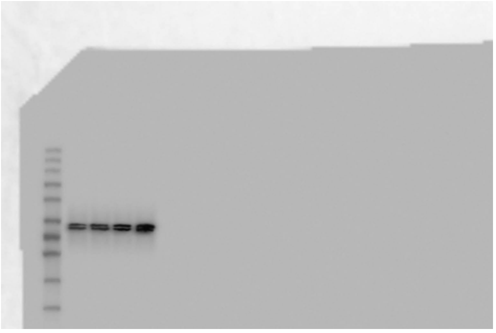

Fig 5j-RBFOX3

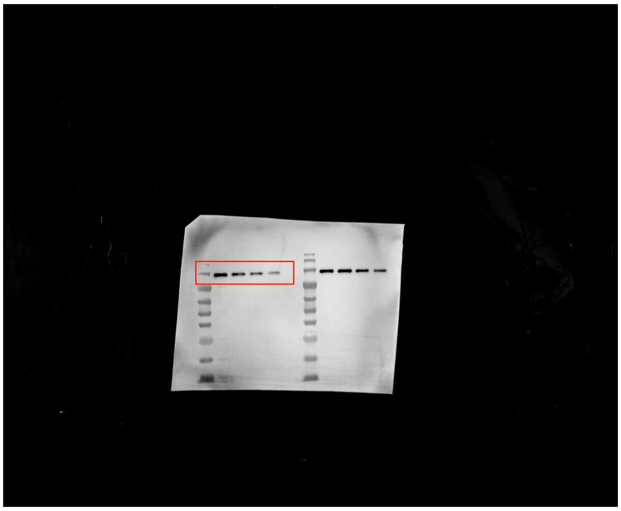

Fig 5q-CTNNB1

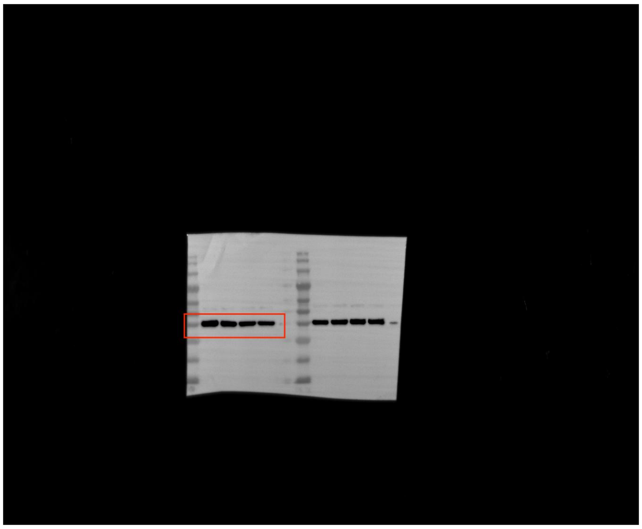

Fig 5q-GAPDH

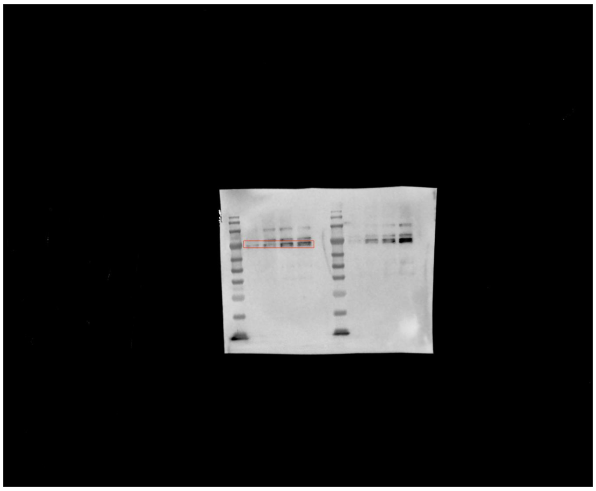

Fig 5q-VANGL2

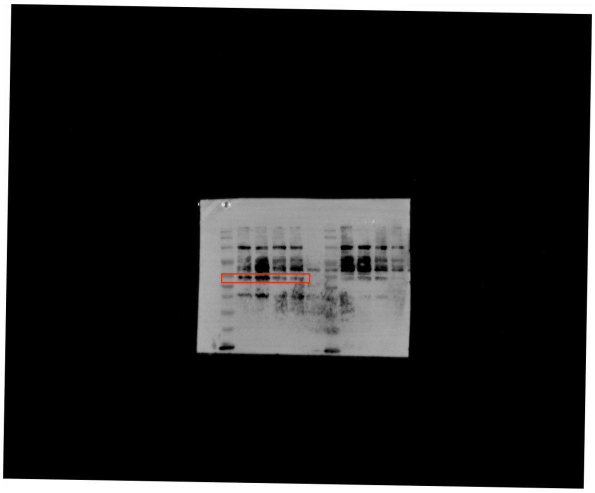

Fig 5q-WNT1

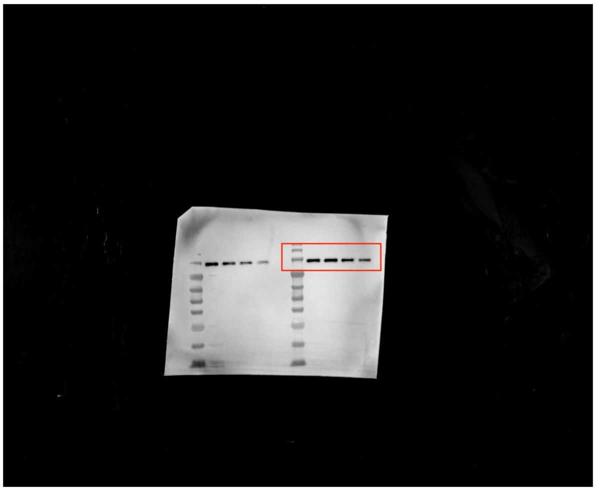

Fig 5u-CTNNB1

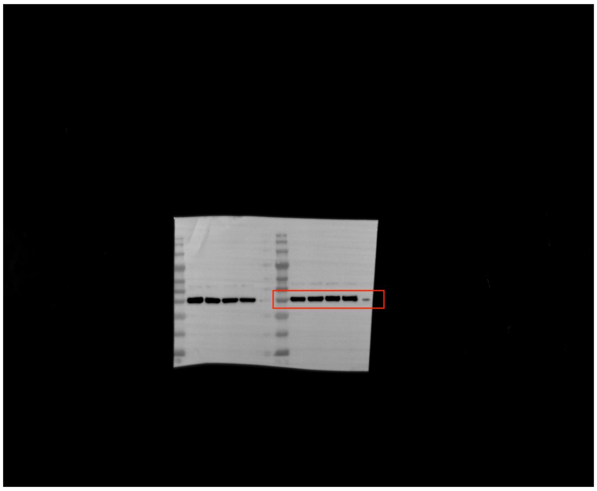

Fig 5u-GAPDH

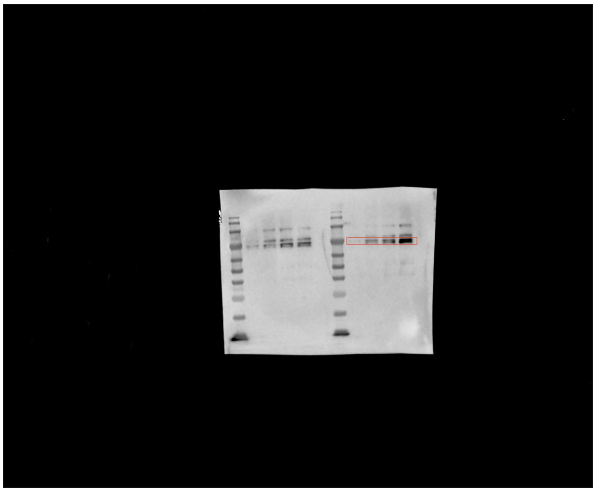

Fig 5u-VANGL2

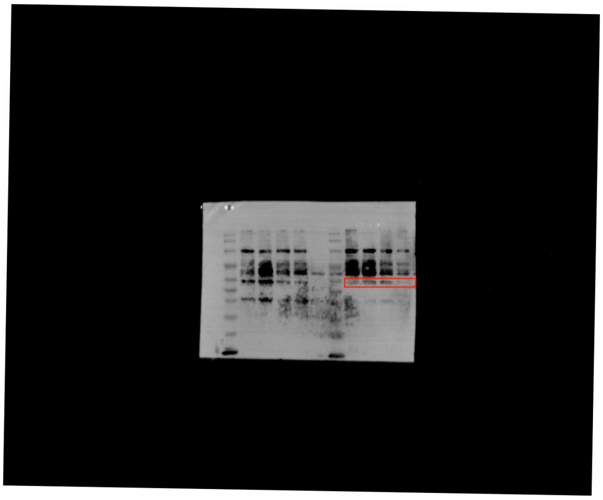

Fig 5u-WNT1

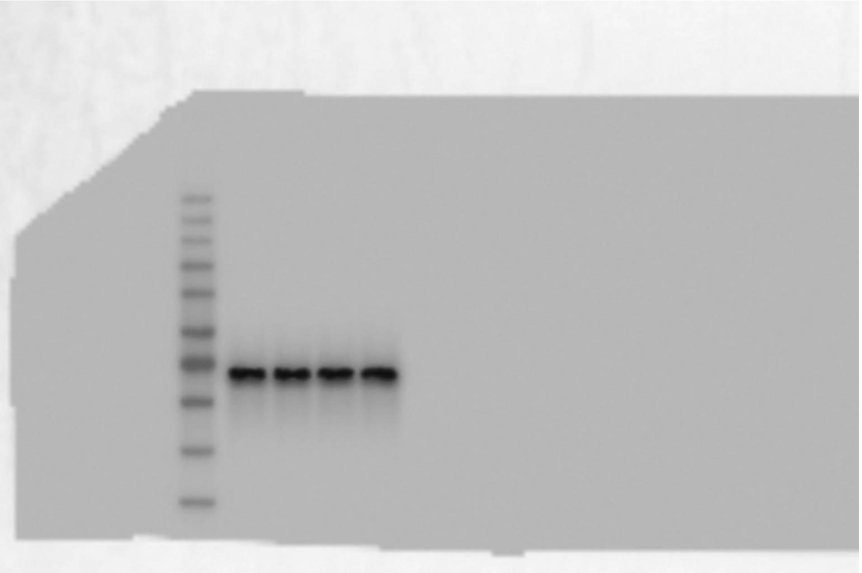

Fig 6k-GAPDH

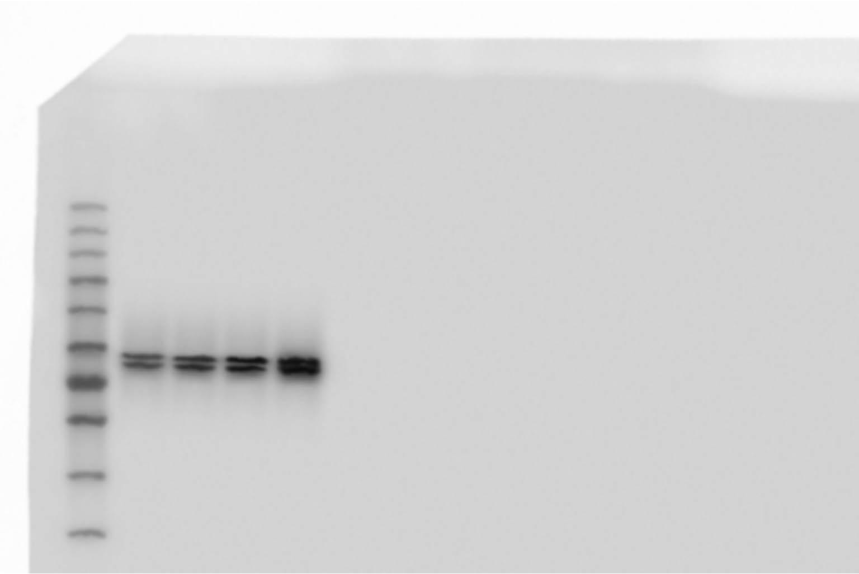

Fig 6k-RBFOX3

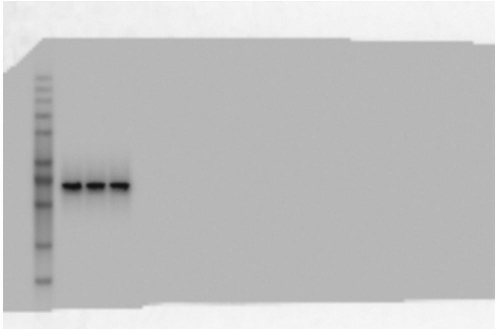

Fig S2a-GAPDH

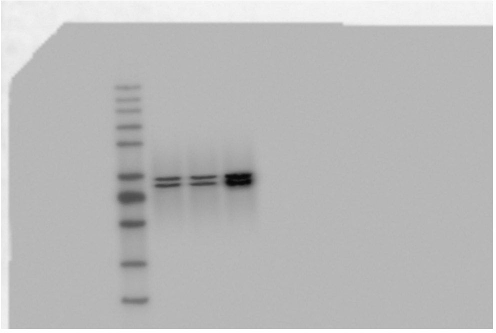

Fig S2a-RBFOX3

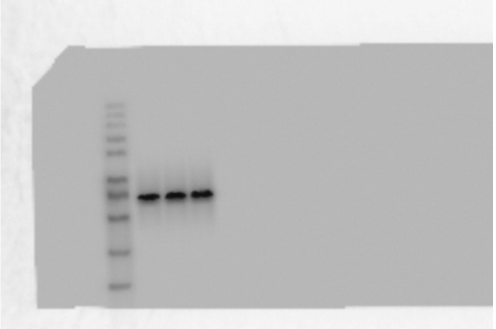

Fig S2b-GAPDH

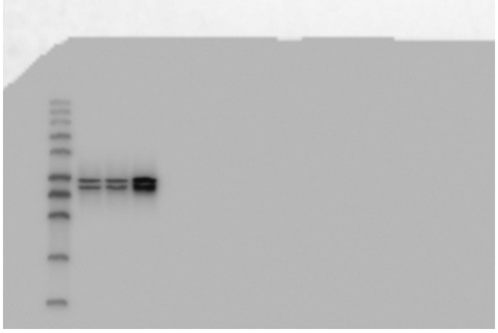

Fig S2b-RBFOX3

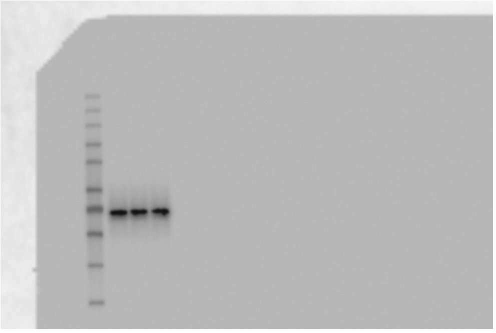

Fig S2g-GAPDH

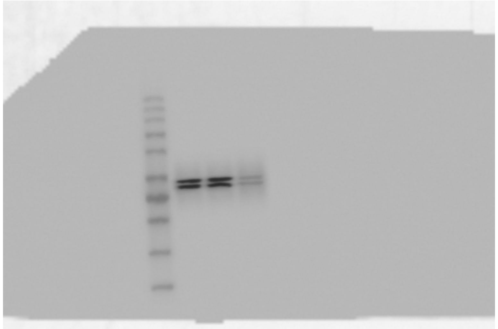

Fig S2g-RBFOX3

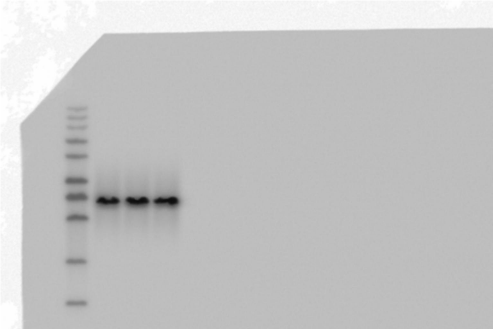

Fig S2h-GAPDH

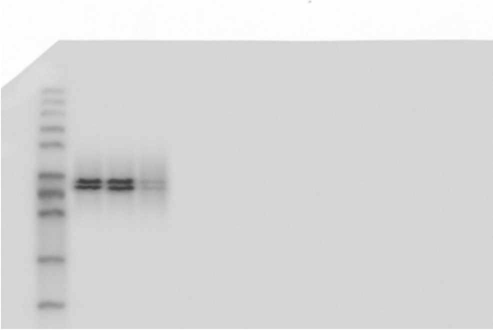

Fig S2h-RBFOX3

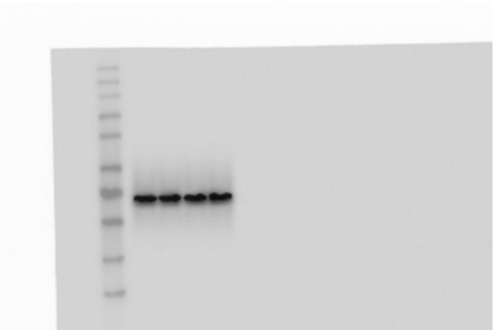

Fig S3a-GAPDH

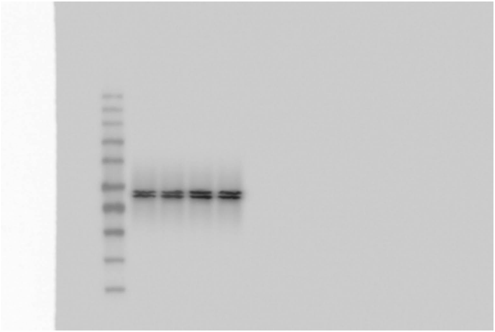

Fig S3a-RBFOX3

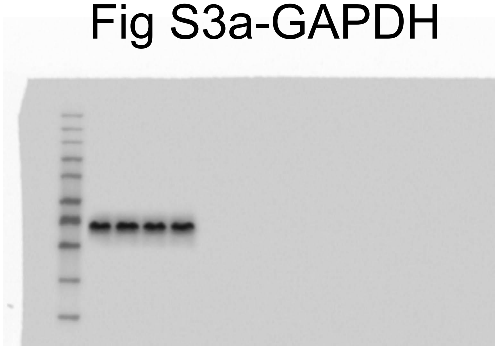

Fig S3b-GAPDH

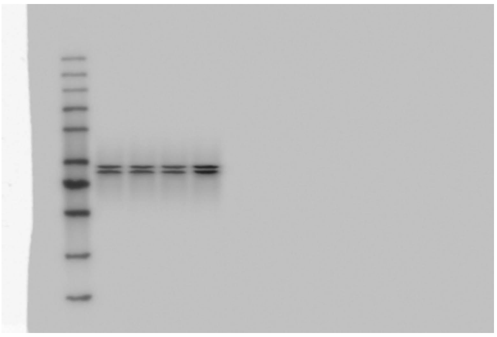

Fig S3b-RBFOX3

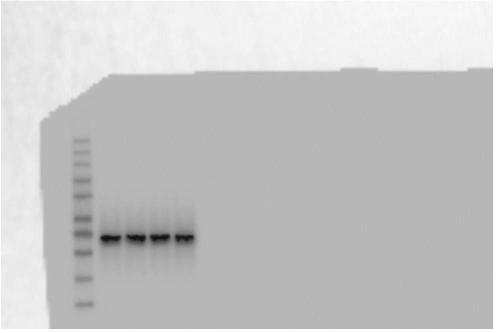

Fig S3c-GAPDH

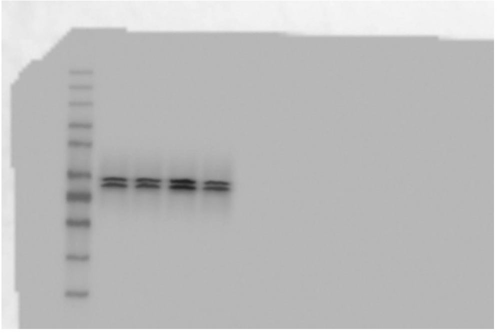

Fig S3c-RBFOX3

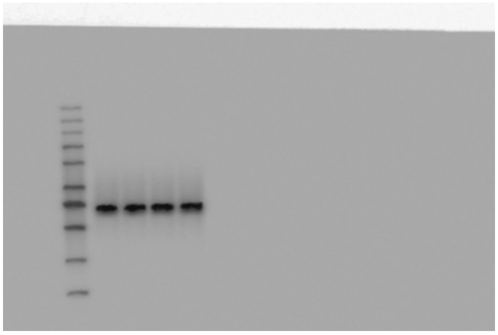

Fig S3d-GAPDH

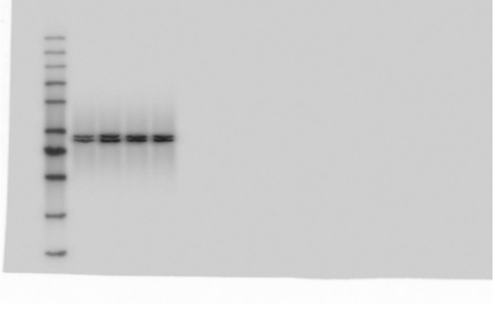

Fig S3d-RBFOX3

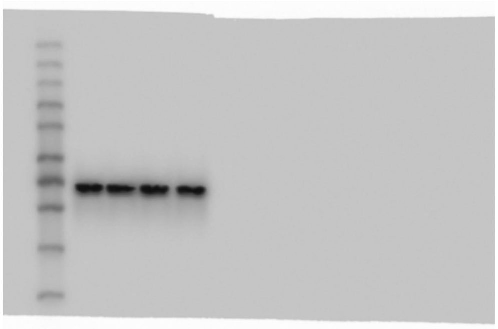

Fig S3e-GAPDH

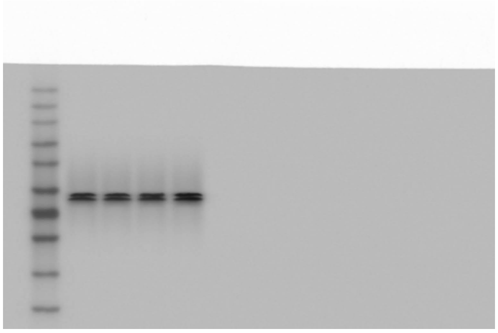

Fig S3e-RBFOX3

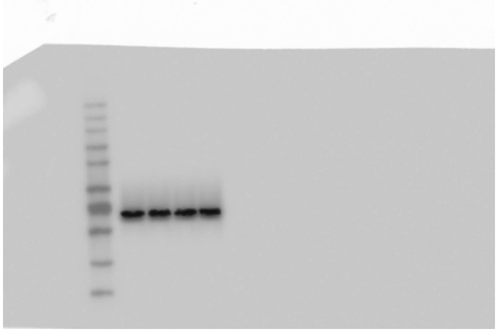

Fig S3f-GAPDH

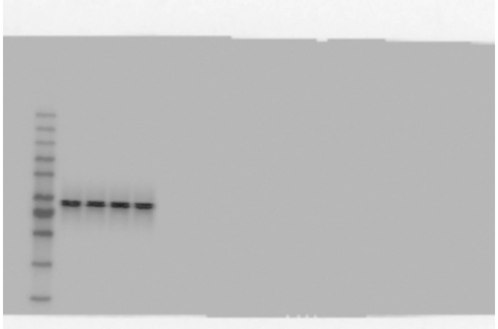

Fig S3f-RBFOX3

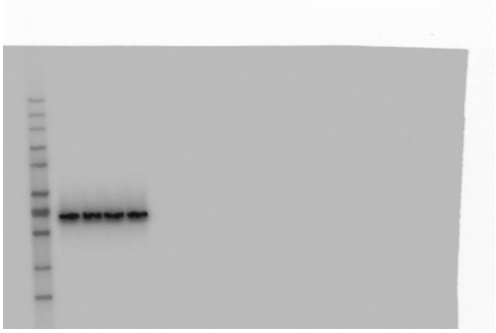

Fig S3g-GAPDH

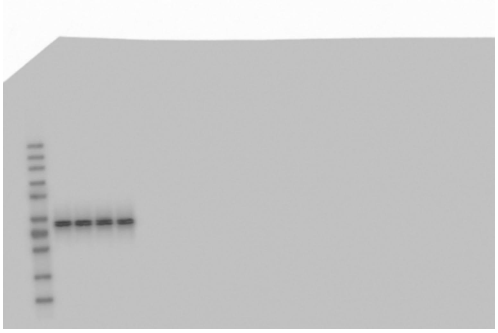

Fig S3g-RBFOX3

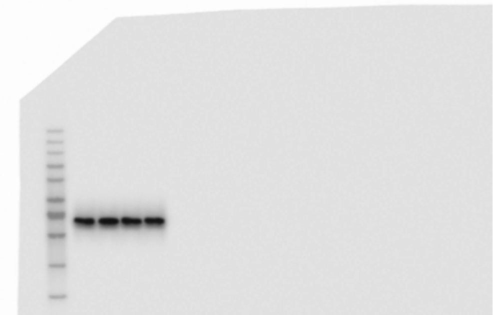

Fig S3h-GAPDH

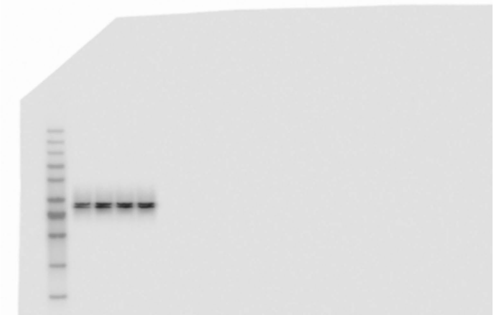

Fig S3h-RBFOX3
